# Blue light attenuates TGF-β2-induced epithelial-mesenchymal transition in human lens epithelial cells via autophagy impairment

**DOI:** 10.1186/s12886-022-02691-6

**Published:** 2022-11-28

**Authors:** Dongyan Zhang, Hong Zhu, Xin Yu, Liyin Wang, Yingying Wen, Liyue Zhang, Jianping Tong, Ye Shen

**Affiliations:** 1 Department of Ophthalmology, Shaoxing Central Hospital, Shaoxing, Zhejiang Province China; 2 Department of Ophthalmology, the First Affiliated Hospital of Zhejiang University, Qingchun Road No.79, Hangzhou, 310003 Zhejiang Province China

**Keywords:** Human lens epithelial cells, Blue light, Epithelial-mesenchymal transition, Autophagy

## Abstract

**Background:**

Pathogenesis of posterior capsular opacification (PCO) was related to pathological epithelial-mesenchymal transition (EMT) of lens epithelial cells (LECs). It has been reported that blue light could have an effect on EMT. This study aims to elucidate the role and potential mechanism of autophagy in EMT after blue light exposure in LECs.

**Methods:**

HLE-B3 cells were treated with TGF-β2 with different concentration and time to induce EMT as a model of PCO in vitro. Cells were exposed to blue light with or without TGF-β2. The expression levels of EMT-associated markers were analyzed by qRT-PCR, western blotting and cell migration ability was determined by transwell migration assay and wound healing assay. The expressions of autophagy-related proteins were analyzed by western blotting, immunofluorescence and transmission electron microscopy. Rapamycin and chloroquine were utilized in cells for autophagy activation and inhibition.

**Results:**

TGF-β2 induced autophagy activation during EMT progression in HLE-B3 cells in a dose- and time-dependent manner. Blue light exposure inhibited TGF-β2-induced EMT characterized by inhibited expression of EMT related markers and reduced migration capacity. Meanwhile, blue light exposure impaired autophagy activated by TGF-β2. Furthermore, Autophagy activation with rapamycin rescued EMT attenuated by blue light. Autophagy inhibition with chloroquine reduced TGF-β2-induced EMT in HLE-B3 cells.

**Conclusion:**

Blue light exposure had inhibited effects on TGF-β2-induced EMT in LECs through autophagy impairment, which provides a new insight on prevention and treatment of PCO.

**Supplementary Information:**

The online version contains supplementary material available at 10.1186/s12886-022-02691-6.

## Background

Posterior capsule opacification (PCO) is the most common complication in cataract patients after extracapsular cataract extraction with or without posterior chamber intraocular lens, especially after then surgery of congenital cataract [1]. In response to surgical trauma, a wound-healing response is initiated in the eye, whereby the residual lens epithelial cells (LCEs) in the anterior capsule undergo inevitable proliferation, migration, and fibrosis, resulting in the formation of PCO. There are mainly two types of PCO: regenerative PCO and fibrous PCO. Regenerative PCO is characterized by hyperplasia and aberrant differentiation of residual LECs at the equator to form lamellar lens masses or Elschning pearls. Fibrotic PCO involves excessive proliferation, migration and invasion, and epithelial-mesenchymal transition (EMT) from residual LECs into fibroblasts in the anterior capsule after surgery, combined with matrix deposition, wrinkling/contraction of the posterior capsule [2]. Abnormal activation of TGF-β2 signaling plays an important role in EMT of cells and tissues. TGF-β can induce the differentiation of epithelial cells into myofibroblasts and then cause lens fibrosis, which is closely related to cell proliferation and apoptosis [3]. Therefore, TGF-β2-induced EMT of LECs is a commonly used in-vitro model for PCO research [4].

Autophagy, a self-protective metabolic process for maintaining cellular homeostasis, ensures the degradation of damaged organelles and abnormal proteins for the recycling of energy and nutrients [5]. Currently, it is controversial of the role of autophagy on EMT regulation. On the one hand, inhibition of autophagy attenuated TGF-β2-induced EMT in primary LECs of rabbits [6]. Moreover, autophagy participated in for activation of TGF-β/Smad3-dependent signaling, leading to EMT and invasion of hepatocellular carcinoma cells [7]. On the other hand, inhibition of autophagy could promote EMT through ROS/HO-1 pathway in ovarian cancer cells [8]. Overall, there is a crosstalk between autophagy and EMT regulation in LECs, but the exact mechanism remains uninvestigated yet.

It is recognized that the short-wavelength blue component (400–480 nm) in artificial light sources is harmful to eye and circadian clock. Excessive exposure to blue light can affect circadian rhythm and lead to eye diseases, such as dry eye disease, glaucoma, keratitis, macular degeneration, etc. [9, 10]. However, blue light is essential for normal eye growth and refractive development [11]. Blue light exposure also slows myopic shift in experimental animals [12, 13]. In the aspects of cancer research, blue light irradiation led to down-regulation of fibronectin (an EMT marker) in bladder cancer [14]. Blue light irradiation also inhibited the proliferation, migration and EMT process of colorectal cancer cells [15]. Additionally, other investigators found that blue light significantly reduced the induced expression of α-SMA (EMT-related protein) in both normal palmar fibroblasts and Duypuytren's fibroblasts [16]. However, the effect of blue light exposure on EMT in eye diseases has not been elucidated, deserving further exploration. Based on the above reviews, we speculated that blue light might participate in the process of EMT through autophagy regulation.

In this study, we established an in-vitro model of TGF-β2-induced EMT in human lens epithelial cells and then explored the effect of blue light exposure on EMT. Our study suggested that autophagy was involved in the regulation of TGF-β2-induced EMT. Blue light exposure reduced TGF-β2-induced EMT in human lens epithelial cells via autophagy inhibition, while autophagy activation by rapamycin reversed the process of EMT attenuated by blue light. This finding could provide basis and new ideas for the prevention and treatment of PCO.

## Materials and methods

### Cell culture

Human lens epithelial B3 (HLE-B3) cells were donated by Dr. Cui from the Second Affiliated Hospital of Zhejiang University and cultured in MEM (41,500,034, Gibco) supplemented with 10% fetal bovine serum (FBS, 10,099-141C, Gibco), and 1% penicillin/streptomycin (15,140–122, Gibco) at 37℃ in a humified incubator with 5% CO_2_.

### TGF-β2 treatment, blue light exposure, and treatment with autophagy modulating reagents

When cells reached 50 ~ 60% confluence, HLECs-B3 cells were treated with different concentrations of TGF-β2 (0, 2, 5, and 10 ng/mL, Peprotech) for 48 h, and the expression of Fibronectin, N-Cadherin, Vimentin, p62 and LC3 could be detected. Similarly, while 50% confluence, cells were treated with 5 ng/mL TGF-β2, or exposed to blue LED light, or exposed to blue light following treatment with 5 ng/mL TGF-β2 for 48 h. The blue light illuminating systems (Honyar Electrical Co., Hangzhou, China) was installed under the incubator partition with 458 nm wavelength at 0.50 mW/cm^2^. Finally, as an autophagy activator or autophagy inhibitor, rapamycin (200 nM, APExBIO) and chloroquine (CQ, 50 μM, APExBIO) were added to the cells for pre-treatment, in combination with or without TGF-β2 or/and blue light for 48 h.

### Western blot analysis

Total proteins were harvested in ice with RIPA buffer (89,900, Thermo Fisher) supplemented with protease inhibitor cocktail (HY-k0010, MCE) and quantified using BCA assay (P0009, Beyotime). Subsequently, 20 μg of the proteins were electrophoresed by 10% SDS-PAGE (1,610,183, Bio-Rad) and then transferred onto PVDF membranes (IPVH00010, Millipore) using a Trans-Blot Turbo transfer system (Bio-Rad). Membranes were blocked with 5% nonfat milk at room temperature and cut into different strips according to the position of the marker. Then different strips were accordingly incubated with indicated primary antibodies at 4 °C overnight. The next day, membranes were probed with HRP-conjugated secondary antibody (1:5000, 7074P2, CST) at room temperature. Protein bands were visualized using hypersensitive ECL kit (BL523B-2, Biosharp) on a Bio-Rad imaging system and analyzed with ImageJ software. The primary antibodies used were diluted with primary antibody dilution Buffer (P0023A, Beyotime) containing anti-Fibronectin (1:1000, ab32417, Abcam), anti-N-cadherin (1:1000, 13,116, CST), anti-Vimentin (1:1000, 5741, CST), anti-p62/STSQM1 (1:10,000, ab109012, Abcam), and anti-LC3B (1:1000, 3868, CST), anti-GAPDH (1:3000, 5174S, CST).

### RNA extraction and qRT-PCR

Total RNA from cells was extracted using Trizol reagent (9109, Takara), and one-step reverse transcription polymerase chain reaction (RT-PCR) kit (RR036A, Takara) as employed to synthesize cDNA. Fibronectin, N-Cadherin and Vimentin expression was measured using SYBR green reagent (RR091A, Takara) on QuantStudioTM Dx Real-Time PCR System (ABI) and β-actin was designated as an internal reference. Relative gene expression was determined using the 2^−△△Ct^ method.

The following primers were used:

**Table:** 

Gene	Forward (5’ to 3’)	Reverse (5’ to 3’)
*β-actin*	ATTGGCAATGAGCGGTTC	GGATGCCACAGGACTCCA
*Fibronectin*	CGGTGGCTGTCAGTCAAAG	AAACCTCGGCTTCCTCCATAA
*N-cadherin*	AGCCAACCTTAACTGAGGAGT	GGCAAGTTGATTGGAGGGATG
*Vimentin*	AGTCCACTGAGTACCGGAGAC	CATTTCACGCATCTGGCGTTC

### Transwell migration assay

Cell migration and invasion capacities were detected using transwell chambers (3422, Corning). Cells in serum-free medium were seeded into the upper chamber of a 24-transwell plate with 8 μm pore filter. MEM contained 20% FBS was added in the lower chambers. After treatment for 48 h, the cells that moved through the underside of the membrane filter were fixed with 4% paraformaldehyde (PFA, BL539A, Biosharp) and stained using 0.1% crystal violet (C0121, Beyotime). Then, images were photographed under an inverted microscope (Olympus), and the number of migrated cells stained were counted blindly in five random fields.

### Wound healing assay

The LECs were seeded in 6-well plates (5 × 10^5^ cells per well) and then treated as previously described when the cell density reached 90% to 100% confluence. The cell monolayers were scratched with a 200 μl pipette tip, followed by removal of the detached cells and debris with PBS. After cultured in serum-free MEM for 0, 6, 24, and 48 h, the representative images of wounds in each well were captured under a microscope. The length of the remaining wound in each image was measured and analyzed using ImageJ software.

### Immunofluorescent staining

The human LCEs were seeded on 24-well plates (2 × 10^4^ cells per well) with cell-climbing slices. After treatment as previously described, the cells were fixed with 4% PFA and permeabilized with 0.1% Triton X‐100. Then, cells were blocked with 5% BSA for 30 min at room temperature and incubated with indicated primary antibodies in a wet chamber at 4 °C overnight. Slides were then incubated with Alexa Fluor 594-conjugated secondary antibody (1:200, 112–585-003, Jackson ImmunoResearch) at room temperature for 1 h. Subsequently Nuclei were counterstained with DAPI (BL105A, Biosharp) and the cell-climbing slices were sealed. Fluorescence images were acquired using a IX71 microscope (Olympus). The primary antibodies used were following: anti-p62 (1:200, ab109012, Abcam), anti-LC3B (1:100, 3868, CST), anti-Ki67 (1:200, ab15580, Abcam).

### Transmission electron microscopy (TEM) analysis

After fixation overnight with 2.5% glutaraldehyde at 4℃, the treated cells were washed in PBS and post fixed in 1% osmium tetroxide (OsO_4_). The cells were then dehydrated in ethanol and acetone, and embedded in EPON resin. Ultrathin Sects. (60–80 nm) were stained with acetic acid uranium and then collected on naked copper grids to be visualized under a TEM system.

### Cell viability assay

The HLE-B3 cells were cultured at a density of 5,000 cells per well in 96-well plates. After treatment, cell viability was monitored using a CCK8 kit (BS350A, Biosharp) following manufacturer’s instructions. Briefly, the conditional medium was replaced with fresh serum-free media containing 10% CCK-8 and the cells incubated for 1 h at 37℃ in the dark. Absorbance at 450 nm was detected using SpectraMax i3x microplate reader (Molecular Devices).

### 5-Ethynyl-2′-deoxyuridine (EdU) assay

EdU assay (C0071S, Beyotime) was conducted to investigate cell proliferation following manufacturer’s instructions. Briefly, half of the medium was replaced with fresh medium containing 20 μM EdU. Thus, the treated cells were incubated with 10 μM EdU for 2 h at 37 °C and then fixed with 4% PFA at room temperature. Following permeabilized with 0.3% Triton X-100 and washed with PBS, staining reactions were performed in dark for 30 min according to the manufacturer's protocol. Subsequently cells were incubated with Hoechst 33,342 for 10 min at room temperature. The EdU positive cells were visualized under a fluorescence microscope (Olympus) and then analyzed using ImageJ software.

### Statistical analysis

All data are presented as the mean ± SD of at least three independent experiments. Data analysis was done for variance on GraphPad Prism 8.0 and SPSS25.0 software. Multiple group comparisons were analyzed by one-way analysis of variance. *P* < 0.05 was considered to indicate a statistically significant difference.

## Results

### TGF-β2 induces EMT and autophagy activation in HLE-B3 cells

**Fig. 1:**
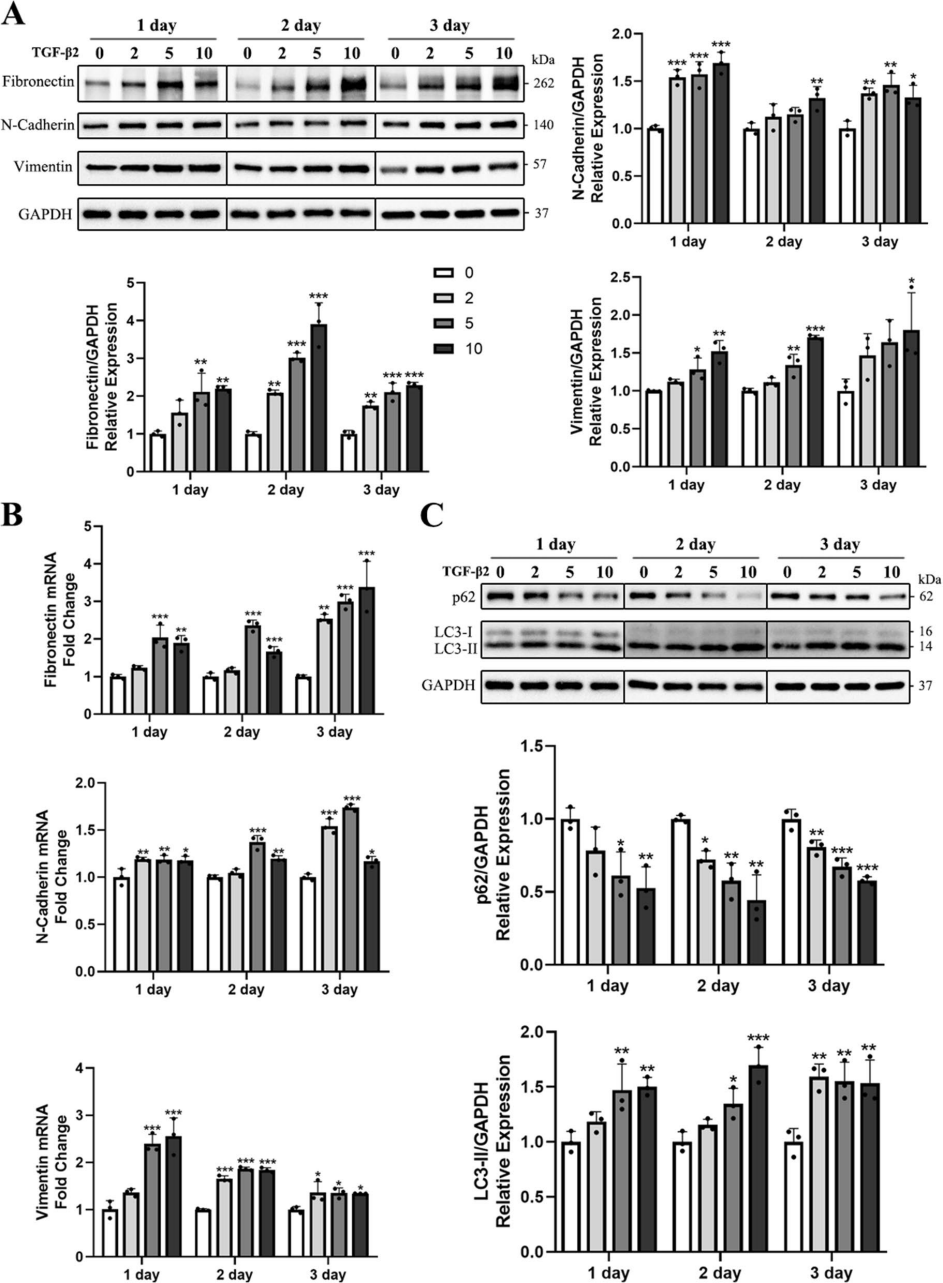
TGF-β2 induces EMT and autophagy activation in HLE-B3 cells. **A** HLE B3 cells were treated with different concentrations (0 ng/ml, 2 ng/ml, 5 ng/ml, 10 ng/ml) of TGF-β2 for 1 day, 2 days and 3 days respectively. The expressions of Fibronectin, N-Cadherin and Vimentin were detected by Western blot analysis and quantified by imageJ software. **B** mRNA expression of EMT markers was determined by qRT-PCR. Data were presented as fold change relative to controls. **C** Western blot analysis showed the effects of TGF-β2 with different concentrations on the expression of p62 and LC3 and relative band intensity of autophagy-associated markers was measured. All the results were presented as mean ± SD. *P < 0.05, **P < 0.01, and ***P < 0.001

To investigate whether the autophagy levels could be influenced during TGF-β2-induced EMT in human LECs, HLE-B3 cells were treated with different concentrations (0 ng/ml, 2 ng/ml, 5 ng/ml, 10 ng/ml) of TGF-β2. The mRNA and protein levels of EMT markers were assessed by western blot and qRT-PCR after treatment for 1 day, 2 days and 3 days respectively. The protein levels of Fibronectin, N-Cadherin and Vimentin were up-regulated obviously over time in TGF-β2-treated groups and the mRNA levels of Fibronectin, N-Cadherin and Vimentin were also elevated (Fig. 1A-B). Indicating that EMT was induced by TGF-β2 treatment in a time- and dose-dependent manner. Additionally, western blot analysis for autophagy markers showed that LC3-II was elevated and p62 was significantly reduced in TGF-β2-treated groups, indicating activation of autophagy flux during TGF-β2 induced EMT in a dose-dependent manner (Fig. 1C).

### Blue light exposure inhibits TGF-β2-induced EMT in HLE-B3 cells

**Fig. 2:**
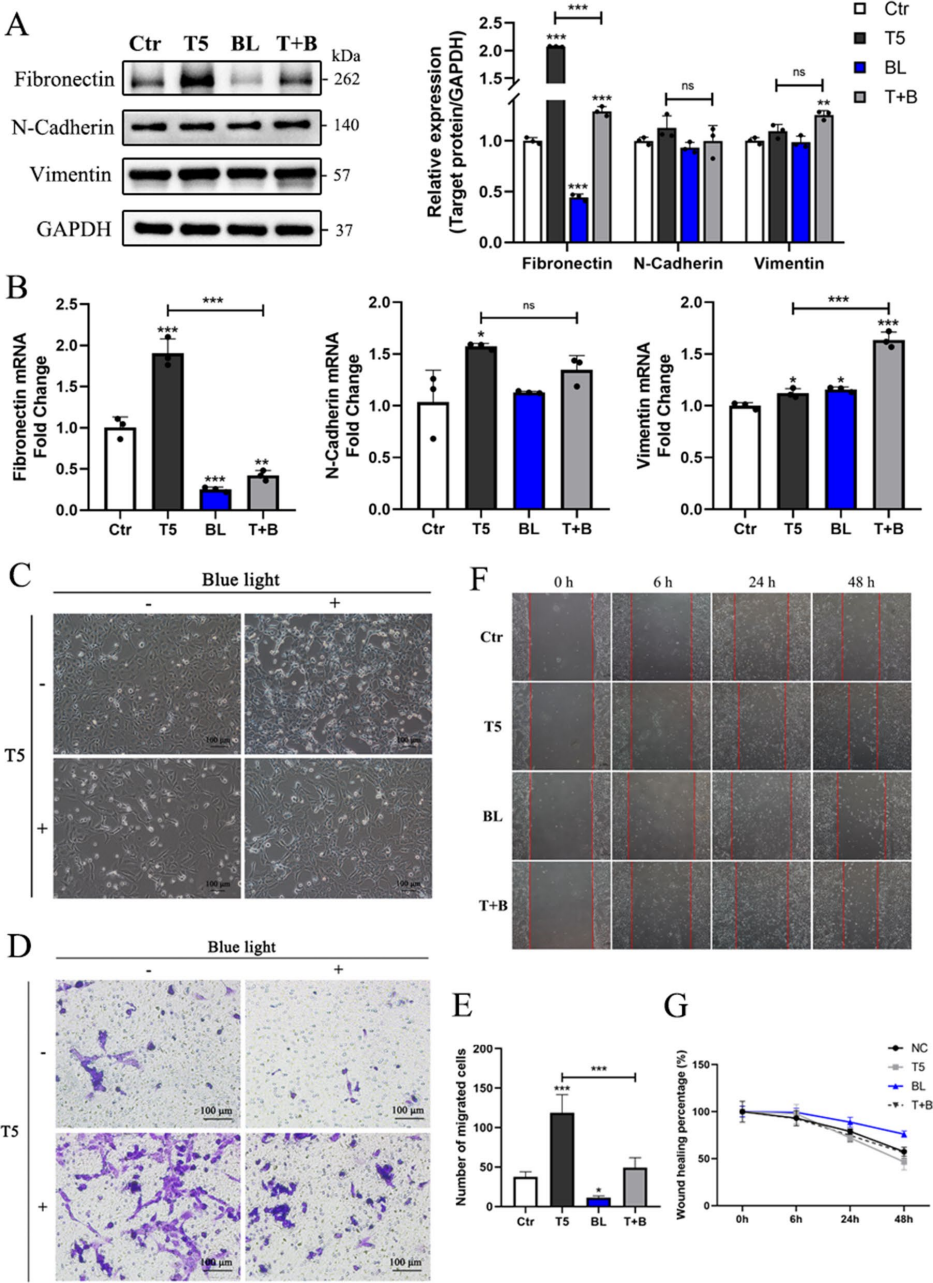
Blue light exposure inhibits TGF-β2-induced EMT in HLE-B3 cells. **A** HLE B3 cells were treated with 5 ng/ml TGF-β2 in the presence or absence of blue light exposure for 48 h. EMT markers were detected by Western blot analysis and densitometry was quantified. **B** qRT-PCR analysis of EMT markers after blue light exposure with or without TGF-β2. **C** Bright field optical microscope images of treated HLE-B3 cells. **D** Representative images of migrated cells in tower chambers following transwell migration assays after treatments. **E** Quantification of migrated cells. **F** Cell motility of treated HLE-B3 cells shown by wound-healing assay. **G** The data of wound-healing assay were quantified based on the percentage of the remaining wound length (the wound length at 0 h was considered 100%). All data were shown as mean ± SD. *P < 0.05, **P < 0.01, and ***P < 0.001

To confirm whether blue light exposure could influence TGF-β2-induced EMT process in LECs, HLE-B3 cells were exposed to blue LED light combined with 5 ng/ml TGF-β2 for 48 h. Compared with the T5 group, the expression and mRNA levels of Fibronectin in T + B group decreased significantly, and the expression and mRNA levels of N-Cadherin in T + B group decreased but it was not statistically significant, while Vimentin mRNA level increased (Fig. 2A-B). Additionally, LECs undergoing EMT acquire a mesenchymal phenotype that allowing them to adopt a migratory and invasive behavior along with morphological changes. Spindle cells during typical EMT process were observed after TGF-β2 treatment (Fig. 2C). In combination with blue light exposure, spindle cells decreased while cuboidal cells increased (Fig. 2C). Subsequently, transwell migration and wound healing assay were performed to further determine the cell migration capacity. TGF-β2 stimulation strongly activated cell migration after 48 h incubation compared with the control group (Fig. 2D-E). Blue light exposure significantly inhibited cell migration activated by TGF-β2 (Fig. 2D-E). Additionally, the wound healing assay revealed that blue light slowed down TGF-β2-induced migration acceleration but there is no statistical significance (Fig. 2F-G). Based on these results, it was demonstrated that blue light exposure markedly inhibited TGF-β2-induced EMT in HLE-B3 cells.

### Blue light exposure impairs autophagy activated by TGF-β2 in HLE-B3 cells

**Fig. 3:**
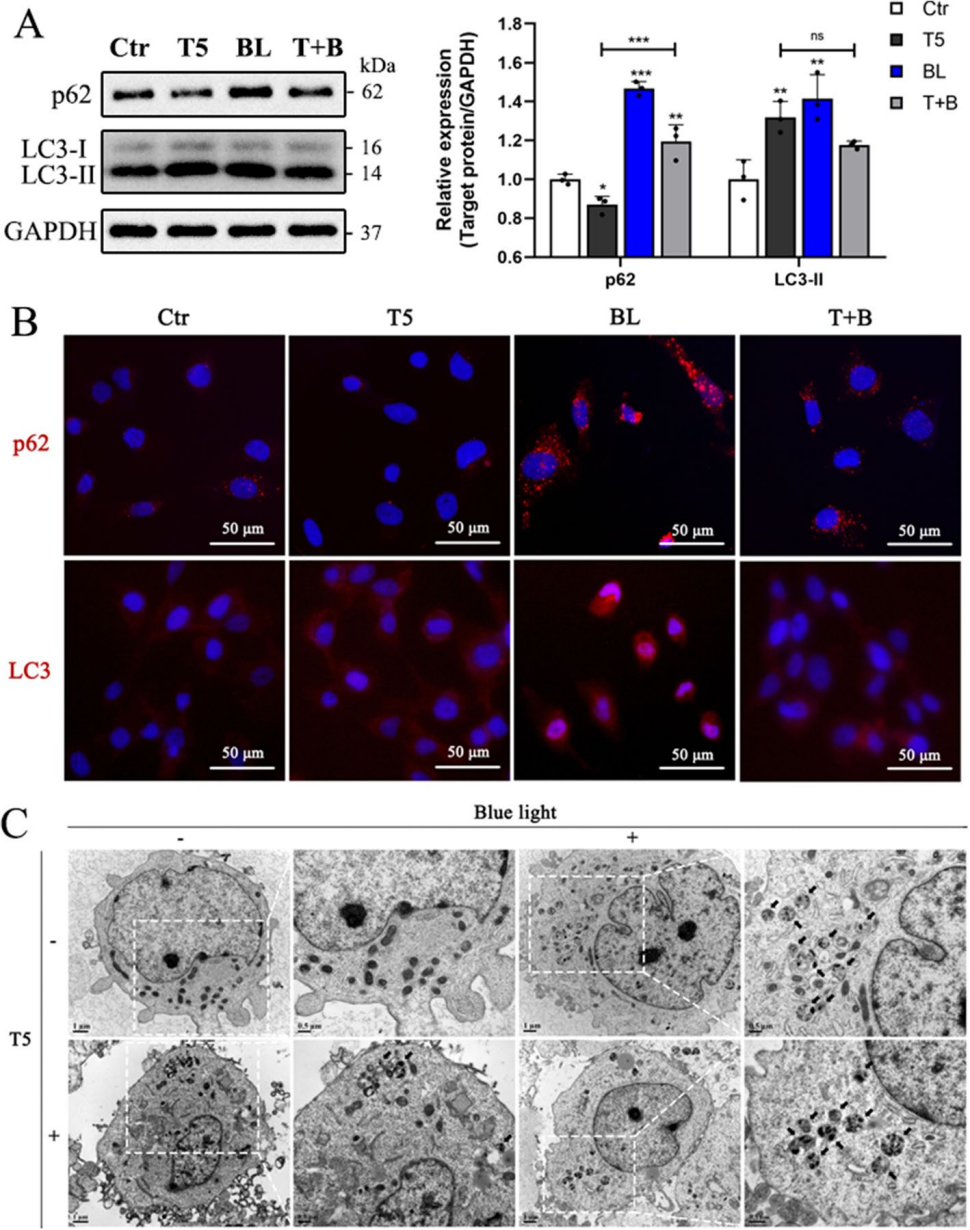
Blue light exposure impairs autophagy activated by TGF-β2 in HLE-B3 cells. **A** Western blot analysis presented the expressions of autophagy markers after treatment with TGF-β2 in the presence or absence of blue light exposure for 48 h. **B** Immunofluorescent staining of p62 and LC3 (red) in HLE-B3 cells after treatments above, in which the nuclei were labeled with DAPI (blue). **C** Autolysosomes (black arrows) containing various components was shown in treated HLE-B3 cells using transmission electron microscopy. Data were presented as mean ± SD. *P < 0.05, **P < 0.01, and ***P < 0.001

To clarify the role of autophagy during the inhibition of TGF-β2-induced EMT under blue light exposure in LECs, the expressions levels of the autophagy markers p62 and LC3 were assessed by western blot and immunofluorescence staining. After exposure to blue light, both p62 and LC3-II were significantly elevated in contrast to the control group, indicating impaired autophagy in HLE-B3 cells (Fig. 3A). P62 was significantly elevated in the T + B group relative to the T5 group, while there was no significant difference in LC3-II (Fig. 3A). Additionally, immunofluorescence staining also revealed the accumulation of p62 puncta and LC3 puncta after blue light exposure (Fig. 3B). Consistent with previous results, p62 was degraded and LC3 puncta was increased during TGF-β2-induced EMT. In combination with blue light exposure, p62 puncta increased slightly in contrast to the T5 group, suggesting that blue light inhibited autophagy activated by TGF-β2. TEM showed markedly characteristic autolysosome in HLE-B3 cells stimulated with TGF-β2 or/and blue light exposure but not in control cells (Fig. 3C). Collectively, we confirmed that blue light exposure impaired autophagy during the inhibition of TGF-β2-induced EMT in HLE-B3 cells.

### Blue light exposure inhibits proliferation of HLE-B3 cells

**Fig. 4:**
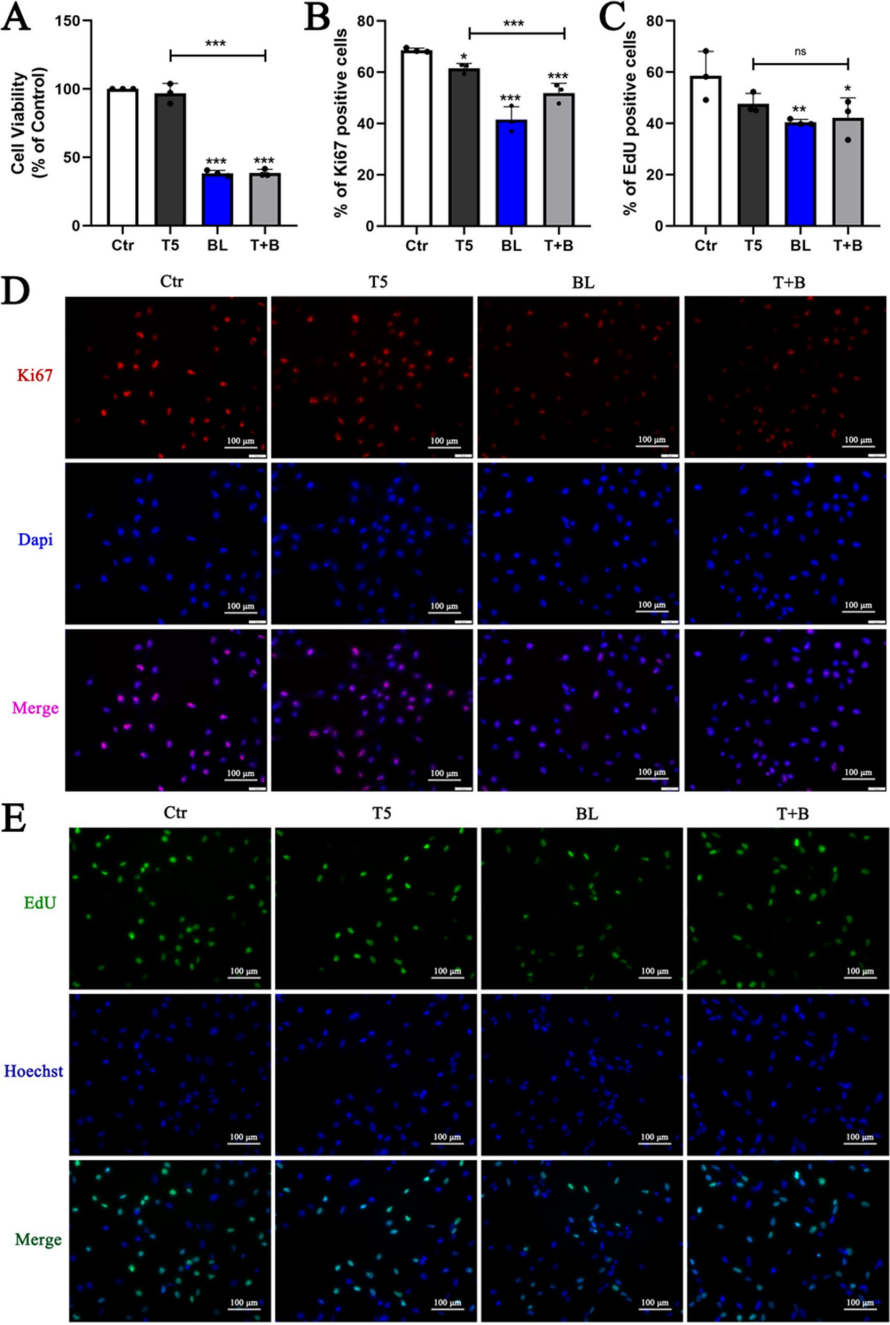
Blue light exposure inhibits proliferation of HLE-B3 cells. **A** Results of the CCK-8 assay showing cell viability measured after 48 h of blue light exposure with or without TGF-β2. **B**, **D** Immunofluorescent staining observed ki67 (red, D) in treated HLE-B3 cells and the percentage of ki67 positive cells (**B**) was measured and quantified by imageJ software. **C**, **E** Representative fluorescence images showing proliferating cells (green, E) after treatments using EdU assay and the percentage of EdU positive cells (**C**) was quantified by imageJ software. All data were shown as mean ± SD. *P < 0.05, **P < 0.01, and ***P < 0.001

To determine the influence on cell proliferation upon TGF-β2 treatment and blue light exposure, cell viability was evaluated using CCK-8 assay. After exposure to blue light with or without TGF-β2, cell viability was significantly decreased relative to the control group (Fig. 4A). Immunofluorescence staining presented that the ki67 positive cells was reduced significantly in the BL group and T + B group, and slightly in the T5 group (Fig. 4B, D). Additionally, EdU-labeled cells in proliferation were greatly reduced in the BL group and T + B group (Fig. 4C, E). These results imply an inhibition of proliferation in HLE-B3 cells exposed to blue light.

### Autophagy activation enhances TGF-β2-induced EMT in HLE-B3 cells

**Fig. 5:**
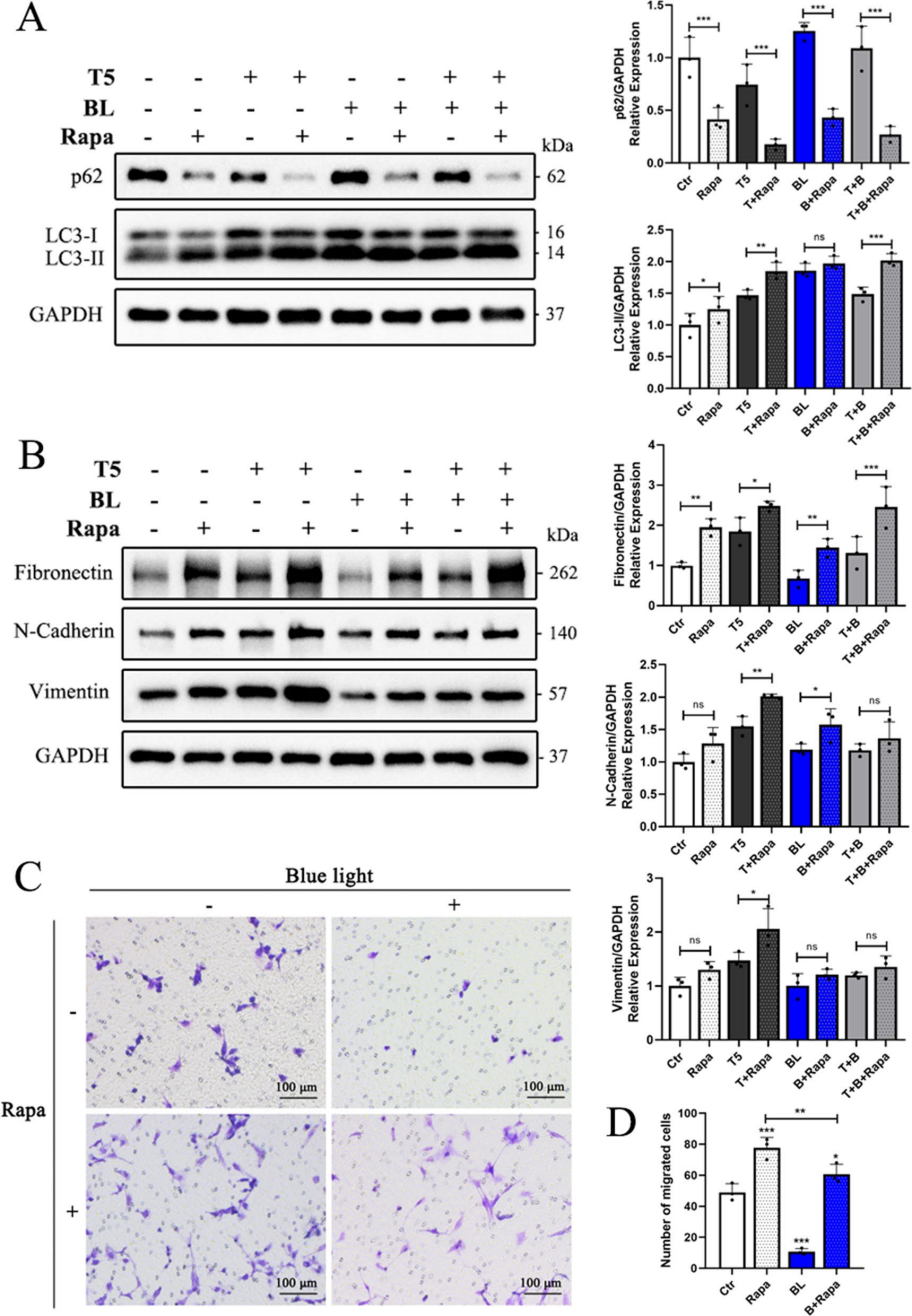
Autophagy activation enhances TGF-β2-induced EMT in HLE-B3 cells. **A**-**B** Western blot analysis showed the expressions of autophagy markers (**A**) and EMT markers (**B**) after treatments with 200 nM rapamycin in the presence or absence of TGF-β2 or/and blue light exposure for 48 h. **C** Cell migration abilities were determined by transwell migration assays after administration of rapamycin, TGF-β2 and blue light exposure. **D** Number of migrated cells in transwell migration assays was quantified. Data were presented as mean ± SD. *P < 0.05, **P < 0.01, and ***P < 0.001

To further gain an insight into the role of autophagy activation in TGF-β2-induced EMT and whether autophagy activation could rescue EMT attenuation induced by blue light, HLE-B3 cells were cultured with rapamycin (an autophagy activator) in the presence or absence of TGF-β2 or/and blue light for 48 h. Rapamycin effectively reduced p62 levels and increased the expression of LC3-II, demonstrating its effect on autophagy activation (Fig. 5A). Moreover, co-treatment of rapamycin and TGF-β2 further promoted TGF-β2-induced elevation of Fibronectin, N-Cadherin, and Vimentin (Fig. 5B). On the contrary, co-treatment of rapamycin and blue light exposure rescued blue light-induced decreases in Fibronectin and N-Cadherin, indicating that the activation of autophagy could further promote the process of EMT (Fig. 5B). Compared with T + B group, the expression of Fibronectin in HLE-B3 cells in T + B + Rapa group increased significantly, while N-Cadherin and Vimentin increased slightly but there was no statistical difference (Fig. 5B). In addition, as shown in Fig. 5C-D, transwell migration assay also suggested that rapamycin significantly accelerated the migration in HLE-B3 cells, reversing the migration slowdown induced by blue light. Hence, autophagy activation reversed the process of EMT attenuated by blue light exposure.

### Autophagy inhibition attenuates TGF-β2-induced EMT in HLE-B3 cells

**Fig. 6:**
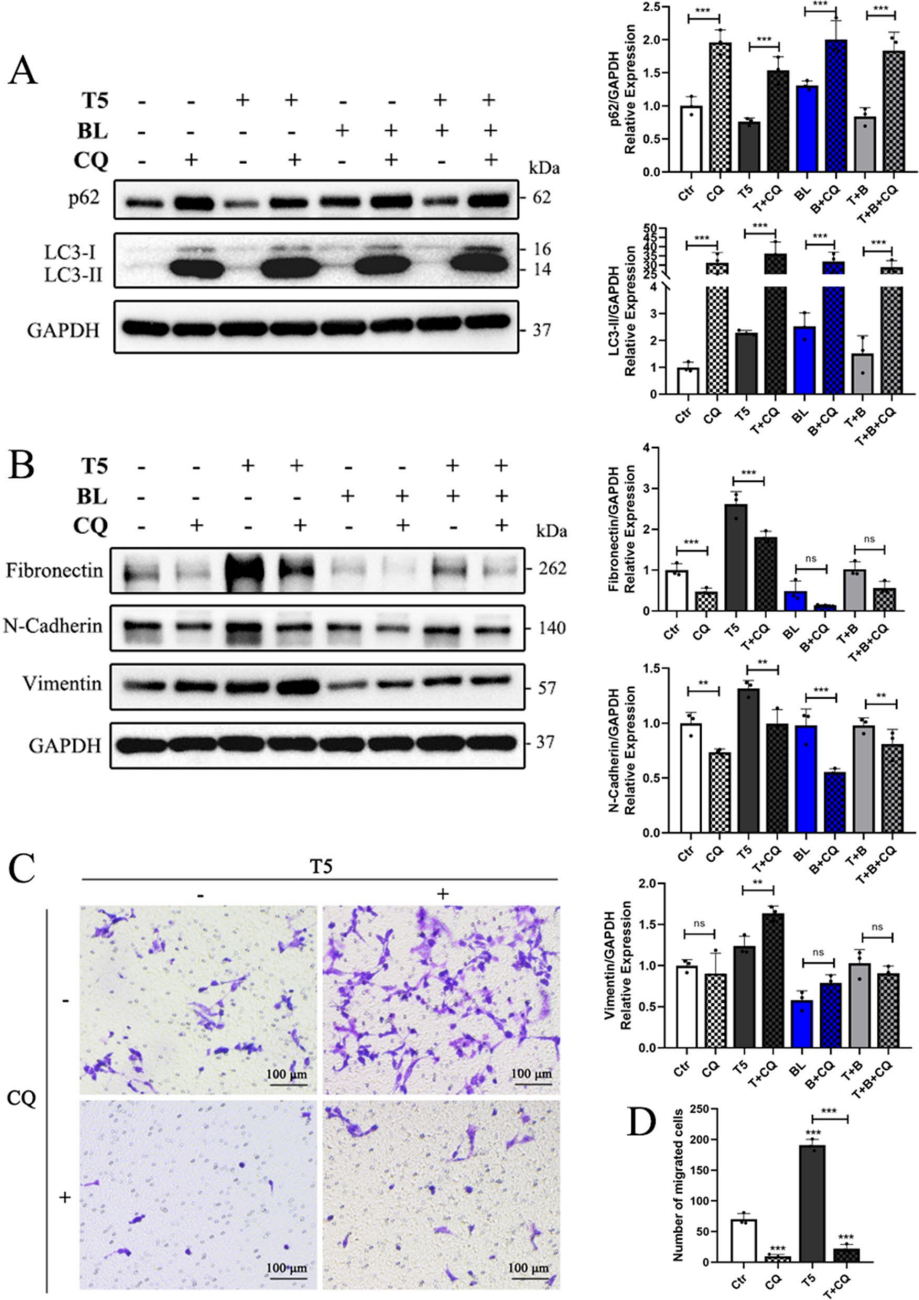
Autophagy inhibition attenuates TGF-β2-induced EMT in HLE-B3 cells. **A**-**B** the expressions of autophagy markers (**A**) and EMT markers (**B**) was detected by western blot analysis after treated with 50 uM CQ in the presence or absence of TGF-β2 or/and blue light exposure for 48 h. (**C**) Cell migration abilities were determined by transwell migration assays after stimulation with CQ, TGF-β2 and blue light exposure. **D** Quantification of migrated cells after transwell migration assays. Data were shown as mean ± SD. *P < 0.05, **P < 0.01, and ***P < 0.001

Given that results above have shown the interplay between autophagy activation and EMT, CQ (an autophagy inhibitor) was added in medium of HLE-B3 cells in the presence or absence of TGF-β2 or/and blue light for 48 h to further confirm that autophagy inhibition could regulate EMT. As Fig. 6A revealed, both p62 and LC3-II obviously elevated in CQ treated groups, suggesting effective inhibition of autophagy by CQ. Besides, compared to the TGF-β2-treated group, there was a remarkable decrease in Fibronectin, N-Cadherin and Vimentin when treated together with CQ (Fig. 6B). Co-treatment with blue light exposure and CQ markedly reduced N-Cadherin levels compared to stimulation with blue light exposure alone (Fig. 6B). Furthermore, CQ significantly retarded the TGF-β2-induced increase of HLE-B3 cells migration (Fig. 6C-D). Based on these findings, it was confirmed that autophagy inhibition could attenuate TGF-β2-induced EMT. Therefore, blue light might attenuate TGF-β2-induced EMT through autophagy inhibition.

## Discussion

In previous studies, high-energy, short-wavelength blue light was always harmful to the eye and biological rhythms [9, 17]. Hence, blue light filtering intraocular lenses (IOLs) have been used after extracapsular cataract extraction surgery to protect macular health and reduce retinal photoxicity induced by blue light [18]. However, the sensitivity to the effects was much different for various types of cells. Our findings strongly supported that 458 nm blue light attenuated TGF-β2-induced EMT in LECs, which was consistent with findings in a previous study that blue light irradiation inhibited cell proliferation, migration and EMT process in colorectal cancer [15]. Therefore, in order to reduce the probability of PCO after cataract extraction surgery, we speculated that partial blue light exposure in the eye was necessary.

Dysregulation of autophagy is related to many lens dysfunctions, such as congenital cataract, age-related cataract, and PCO [19–22]. Previous findings have shown that sulforaphane reduced growth, migration, and viability in lens cells through ER stress and autophagy upon reactive oxygen species (ROS) production, and thus could serve as a putative therapeutic agent for PCO [23]. Compared with rapamycin, PP242 (a new-generation mTOR inhibitor) strongly inhibited the crucial cellular events in the formation of PCO through induction of autophagy and apoptosis, involving proliferation, attachment, and migration [24]. Additionally, it is worth mentioning that the production of large amounts of TGF-β2 in response to cataract surgical stimulation can induce EMT progression in residual LECs with enhanced migration capability, therefore indicating the crucial significance in deciphering the mechanism of TGF-β2-induced-EMT for the sake of therapeutic exploration in PCO [3]. As briefly introduced before, the effect of autophagy regulation in the EMT process was ambiguous and there is a complex link between these two processes. On the one side, cells required autophagy activation to survive during the EMT. On the other side, autophagy functions, as an onco-suppressive signal, could hinder the early phases of metastasization and activation of the EMT in cancers [25]. In eye research, autophagy blockage reduced TGF-β2-triggered EMT in LECs, suggesting that it would be a novel therapy method of autophagy inhibition for prevention and treatment of the fibrotic cataract [6]. Hence, in this study, we established a cellar model of TGF-β2-induced EMT in HLE-B3 cells and then investigated the role of autophagy during the inhibition of EMT under blue light exposure for the very first time.

The TGF-β contributes to EMT activation mainly by mediating Smad and non-Smad signal transduction pathways [26]. During the process of EMT, TGF-β can induce accumulation of autophagosomes and activate the autophagy flux upon stimulating the expression of several autophagy-related genes, such as Beclin-1, Atg5, Atg7, and death-associated protein kinase (Dapk) [27]. In this study, expression of Fibronectin, N-cadherin and Vimentin was examined as EMT markers. Fibronectin, a high molecular weight glycoprotein, can bind to integrins in the extracellular matrix (ECM) [28]. N-cadherin, one of cell adhesion molecules, functions in the formation of adherens junctions to bind cells with each other [29]. Vimentin is abundantly expressed in mesenchymal cells as a type III intermediate filament protein [30]. LC3 and p62 are both commonly used as the markers of macroautophagy. During autophagosome formation in autophagy activation, cytoplasmic LC3 (LC3-I) will be enzymatically hydrolyzed and transformed into membrane type (LC3-II) [31]. In addition to LC3, SQSTM1/p62, as an autophagic substrate, is normally degraded during autophagy but accumulates upon autophagy impairment [32]. In the present study, in addition to the rise of autophagosomes under TEM, the conversion of LC3-I to LC3-II and the degradation of p62 was elevated in a dose-dependent manner in HLE-B3 cells after TGF-β2 stimulation, implying that autophagy as a mediator participated in the process of TGF-β2-induced EMT of LECs [5]. The significantly reduced translation and transcription levels of EMT markers and the decreased migration ability after blue light exposure suggested that blue light inhibited cell migration and EMT process induced by TGF-β2. Interestingly, blue light specifically inhibits fibronectin expression. Lipofuscin, whose major bis-retinoid component is A2E, accumulates normally with age and is related to age-related macular degeneration [33]. Mai T et al*.* recently reported that blue light irradiation could cause cleavage throughout the A2E molecule closest to the pyridinium ring, and attached to the fibronectin peptide preferentially at lysine and arginine residues [34]. Meanwhile, blue light exposure further enhanced TGF-β2-induced LC3-II conversion while reversing p62 degradation, leading to p62 accumulation, indicating that impaired autophagy after blue light exposure during the inhibition of TGF-β2-induced EMT. Moreover, the accumulation of autophagolysosomes and LC3-II after blue light exposure might be attributed to reduced autophagolysosome degradation due to blocked autophagosome-lysosome fusion [35]. Overall, according to our data, blue light induced autophagy impairment and alleviated TGF-β2-induced EMT, suggesting that administration of blue light exposure may potentially offer a novel therapeutic approach for the prevention of PCO.

However, Blue light mainly worked when a large amount of extracellular matrix like fibronectin was induced by TGF-β2, while physiological wound healing would not work under low amounts of ECM [36]. We also determined the effect of blue light on the proliferation and cell viability of HLE-B3 cells. CCK8 results showed that blue light reduced the cell viability with or without TGF-β2. The decrease of ki67 positive cells and EdU positive cells suggested that the cell proliferation decreased after blue light exposure. Accordingly, blue light has a certain phototoxicity on lens epithelial cells. Hence, considering the harm of blue light to the ocular surface, lens, and retina, blue light should be carefully applied in the clinical situation.

Then, we further determined the effect of autophagy regulation in EMT attenuated by blue light exposure. Our data showed the increased autophagy flow and a greater transdifferentiation capability from LECs into myofibroblasts by assessing the mesenchymal markers after rapamycin treatment, with or without TGF-β2 combination or/and blue light. Rapamycin also significantly improved the cell migration property, reversing the migration slowdown induced by blue light. Furthermore, chloroquine, as an autophagy inhibitor, reduced the expression of TGF-β2-induced EMT markers and cell migration ability. These results confirmed our conjecture that blue light attenuated TGF-β2-induced EMT through autophagy inhibition. Further studies are needed to elucidate the effects of LED with different wavelengths which may have different effects, and to determine the underlying molecular mechanisms.

In summary, our study provided a direct evidence that blue LED exposure has inhibited effects on TGF-β2-induced EMT in LECs, which characterized by inhibited expression of EMT related markers and reduced migration, and it might be caused by impaired autophagy. Collectively, these findings provided a novel potential therapeutic strategy for PCO in the future.

## Conclusions

Blue light exposure had an inhibited effect on TGF-β2-induced EMT in LECs through autophagy impairment, which provides a new insight on prevention and treatment of PCO.

## Supplementary Information

**Additional file 1.**

## Data Availability

All data generated or analyzed during this study are included in this article. Further enquiries can be directed to the corresponding author.

## Abbreviations

PCO: Posterior capsular opacification
EMT: Epithelial-mesenchymal transition
LECs: Lens epithelial cells
HLE-B3: Human lens epithelial B3 cells
CQ: Chloroquine

## References

[1] Apple DJ, Solomon KD, Tetz MR, Assia EI, Holland EY, Legler UF (1992). Posterior capsule opacification. Surv Ophthalmol.

[2] Wormstone IM, Wormstone YM, Smith AJO, Eldred JA (2021). Posterior capsule opacification: What's in the bag?. Prog Retin Eye Res.

[3] Kubo E, Shibata T, Singh DP, Sasaki H (2018). Roles of TGF beta and FGF Signals in the Lens: Tropomyosin Regulation for Posterior Capsule Opacity. Int J Mol Sci..

[4] de Iongh RU, Wederell E, Lovicu FJ, McAvoy JW (2005). Transforming growth factor-beta-induced epithelial-mesenchymal transition in the lens: a model for cataract formation. Cells Tissues Organs.

[5] Klionsky DJ, Abdel-Aziz AK, Abdelfatah S, Abdellatif M, Abdoli A, Abel S (2021). Guidelines for the use and interpretation of assays for monitoring autophagy (4th edition)(1). Autophagy.

[6] Sun Y, Xiong L, Wang X, Wang L, Chen B, Huang J (2021). Autophagy inhibition attenuates TGF-beta2-induced epithelial-mesenchymal transition in lens epithelial cells. Life Sci.

[7] Li J, Yang B, Zhou Q, Wu Y, Shang D, Guo Y (2013). Autophagy promotes hepatocellular carcinoma cell invasion through activation of epithelial-mesenchymal transition. Carcinogenesis.

[8] Zhao Z, Zhao J, Xue J, Zhao X, Liu P (2016). Autophagy inhibition promotes epithelial-mesenchymal transition through ROS/HO-1 pathway in ovarian cancer cells. Am J Cancer Res.

[9] Ouyang X, Yang J, Hong Z, Wu Y, Xie Y, Wang G (2020). Mechanisms of blue light-induced eye hazard and protective measures: a review. Biomed Pharmacother.

[10] Touitou Y, Point S (2020). Effects and mechanisms of action of light-emitting diodes on the human retina and internal clock. Environ Res.

[11] Rucker F (2019). Monochromatic and white light and the regulation of eye growth. Exp Eye Res.

[12] Rucker F, Britton S, Spatcher M, Hanowsky S (2015). Blue Light Protects Against Temporal Frequency Sensitive Refractive Changes. Invest Ophthalmol Vis Sci.

[13] Wen Y, Jin L, Zhang D, Zhang L, Xie C, Guo D (2021). Quantitative proteomic analysis of scleras in guinea pig exposed to wavelength defocus. J Proteomics.

[14] Shakibaie M, Vaezjalali M, Rafii-Tabar H, Sasanpour P (2019). Synergistic effect of phototherapy and chemotherapy on bladder cancer cells. J Photochem Photobiol B.

[15] Yan G, Zhang L, Feng C, Gong R, Idiiatullina E, Huang Q (2018). Blue light emitting diodes irradiation causes cell death in colorectal cancer by inducing ROS production and DNA damage. Int J Biochem Cell Biol.

[16] Krassovka J, Borgschulze A, Sahlender B, Logters T, Windolf J, Grotheer V (2019). Blue light irradiation and its beneficial effect on Dupuytren's fibroblasts. PLoS ONE.

[17] Tosini G, Ferguson I, Tsubota K (2016). Effects of blue light on the circadian system and eye physiology. Mol Vis.

[18] Downie LE, Busija L, Keller PR (2018). Blue-light filtering intraocular lenses (IOLs) for protecting macular health. Cochrane Database Syst Rev..

[19] Morishita H, Mizushima N (2016). Autophagy in the lens. Exp Eye Res.

[20] Wignes JA, Goldman JW, Weihl CC, Bartley MG, Andley UP (2013). p62 expression and autophagy in alphaB-crystallin R120G mutant knock-in mouse model of hereditary cataract. Exp Eye Res.

[21] Sagona AP, Nezis IP, Stenmark H (2014). Association of CHMP4B and autophagy with micronuclei: implications for cataract formation. Biomed Res Int.

[22] Qin C, Liu S, Wen S, Han Y, Chen S, Qie J (2021). Enhanced PCO prevention of drug eluting IOLs via endocytosis and autophagy effects of a PAMAM dendrimer. J Mater Chem B.

[23] Liu H, Smith AJ, Ball SS, Bao Y, Bowater RP, Wang N (2017). Sulforaphane promotes ER stress, autophagy, and cell death: implications for cataract surgery. J Mol Med (Berl).

[24] Feng H, Yang Z, Bai X, Yang M, Fang Y, Zhang X (2018). Therapeutic potential of a dual mTORC1/2 inhibitor for the prevention of posterior capsule opacification: An in vitro study. Int J Mol Med.

[25] Gugnoni M, Sancisi V, Manzotti G, Gandolfi G, Ciarrocchi A (2016). Autophagy and epithelial-mesenchymal transition: an intricate interplay in cancer. Cell Death Dis.

[26] Zhang C, Zhang X, Xu R, Huang B, Chen AJ, Li C (2017). TGF-beta2 initiates autophagy via Smad and non-Smad pathway to promote glioma cells' invasion. J Exp Clin Cancer Res.

[27] Suzuki HI, Kiyono K, Miyazono K (2010). Regulation of autophagy by transforming growth factor-beta (TGF-beta) signaling. Autophagy.

[28] Pankov R, Yamada KM (2002). Fibronectin at a glance. J Cell Sci.

[29] Leckband D, Prakasam A (2006). Mechanism and dynamics of cadherin adhesion. Annu Rev Biomed Eng.

[30] Usman S, Waseem NH, Nguyen TKN, Mohsin S, Jamal A, Teh MT (2021). Vimentin Is at the Heart of Epithelial Mesenchymal Transition (EMT) Mediated Metastasis. Cancers (Basel)..

[31] Klionsky DJ, Abdelmohsen K, Abe A, Abedin MJ, Abeliovich H, Acevedo Arozena A, et al. Guidelines for the use and interpretation of assays for monitoring autophagy (3rd edition). Autophagy. 2016;12(1):1–222. 10.1080/15548627.2015.1100356 PMC4835977 26799652

[32] Bjorkoy G, Lamark T, Brech A, Outzen H, Perander M, Overvatn A (2005). p62/SQSTM1 forms protein aggregates degraded by autophagy and has a protective effect on huntingtin-induced cell death. J Cell Biol.

[33] Dorey CK, Wu G, Ebenstein D, Garsd A, Weiter JJ (1989). Cell loss in the aging retina. Relationship to lipofuscin accumulation and macular degeneration. Invest Ophthalmol Vis Sci..

[34] Thao MT, Renfus DJ, Dillon J, Gaillard ER (2014). A2E-mediated photochemical modification to fibronectin and its implications to age-related changes in Bruch's membrane. Photochem Photobiol.

[35] Mahli A, Saugspier M, Koch A, Sommer J, Dietrich P, Lee S (2018). ERK activation and autophagy impairment are central mediators of irinotecan-induced steatohepatitis. Gut.

[36] Xue M, Jackson CJ (2015). Extracellular Matrix Reorganization During Wound Healing and Its Impact on Abnormal Scarring. Adv Wound Care (New Rochelle).

