# Supplementary material for: Blue light attenuates TGF-β2-induced epithelial-mesenchymal transition in human lens epithelial cells via autophagy impairment
Source: BMC Ophthalmol. 2022 Nov 28;22:456. doi: 10.1186/s12886-022-02691-6 (PMC9706913; doi:10.1186/s12886-022-02691-6)
Supplement: Supplementary file 1 — Additional file 1. [file 12886_2022_2691_MOESM1_ESM.pdf]

Blue light attenuates TGF-β2-induced epithelial-mesenchymal transition in human lens epithelial cells via autophagy impairment

Dongyan Zhang[1], Hong Zhu[2], Xin Yu[2], Liyin Wang[2], Yingying Wen[2], Liyue Zhang[2], Jianping Tong[2], Ye Shen[2*]

[1] Department of Ophthalmology, Shaoxing Central Hospital, Shaoxing, Zhejiang Province, China.

[2] Department of Ophthalmology, the First Affiliated Hospital of Zhejiang University, Hangzhou, Zhejiang Province, China

[*]Corresponding Author:

Ye Shen

Ophthalmology department, the First Affiliated Hospital of Zhejiang University,

Qingchun Road No.79, Hangzhou, Zhejiang Province, 310003, China

Tel: +86 13605711083

E-mail: idrshen@zju.edu.cn

SENSING Report of Spectroradiometric & Electric Analysis for Light Source

Model No.:
Sample SN:
Manufacturer: Date:
Tested By: Reviewed By: AC

Test Condition

Temperature: °C RH: %
Spectrum Range: 380-780 nm Scan Step: 5 nm

Spectroradiometric Parameters

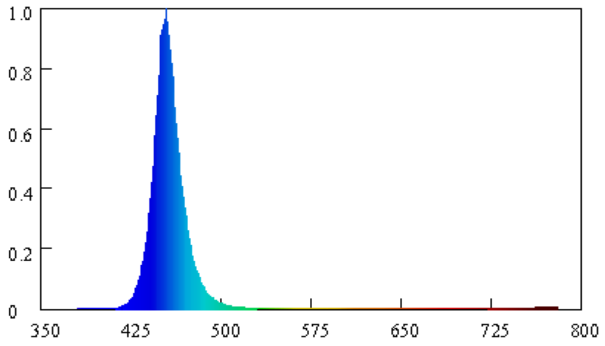

Spectral Distribution

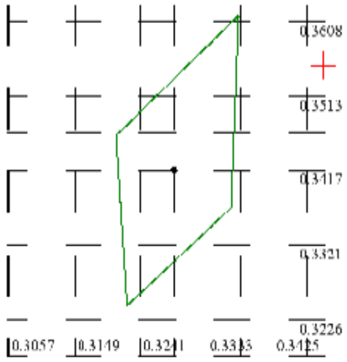

Nominal CCT:LED_5700K
x0=0.3493 y0=0.3552

Chromaticity Coordinates: x=0.1502 y=0.0354 u'=0.1922 v'=0.102

Correlated Color Temperature: 30000 K Dominant Wavelength: 458.0 nm(E)

Rendering Index: Ra=-50.3 Peak Wavelength: 453.9 nm

Purity: 0.9743 Bandwidth: 21.4nm

Luminous Flux: 43.259 lm Radiant Flux: 1.304 W

Color Ratio: Kr=4.0% Kg=7.9% Kb=88.1%

Chromaticity Difference: -0.20441Duv

R1=6 R2=-37 R3=-152 R4=-92 R5=15 R6=-48 R7=-54 R8=-40

R9=-197 R10=-218R11=-118R12=-110R13=-13 R14=-32 R15=21

Electric Parameters

Voltage: 11.42 V Current: 0.5248 A

Power Factor: 1.001 Power: 6.0 W

Luminous Efficacy: 7.21 lm/W

SENSING Instruments Co. Ltd

Fig S1. Report of spectroradiometric and electric analysis for blue LED light Source

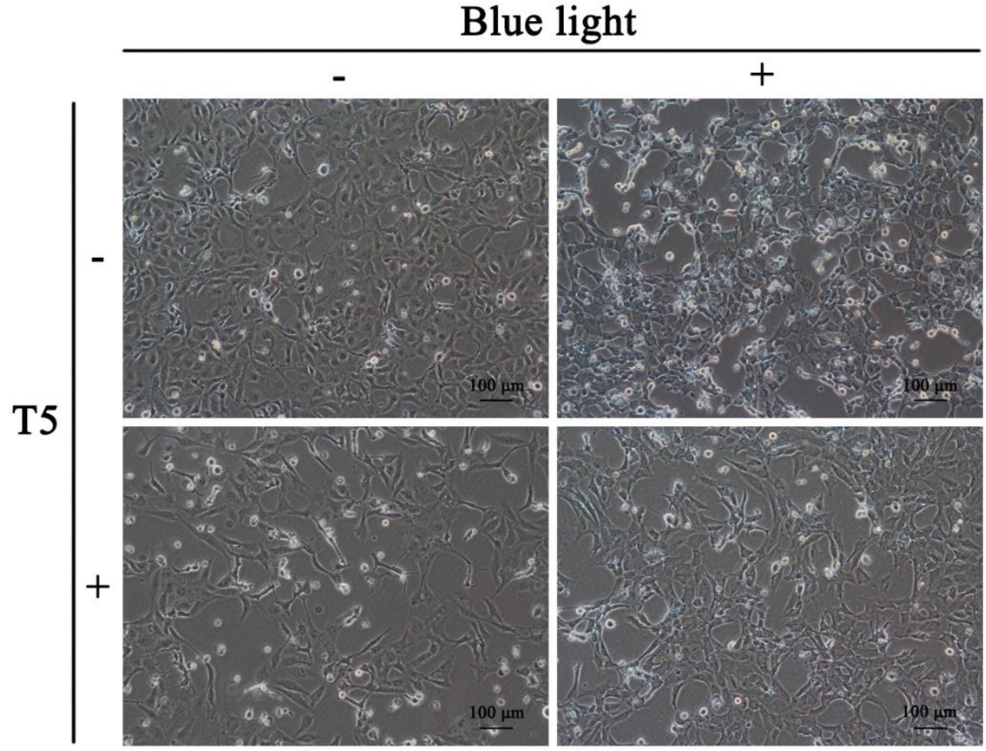

Fig S2. Bright field optical microscope images of treated HLE-B3 cells in Fig 2C.

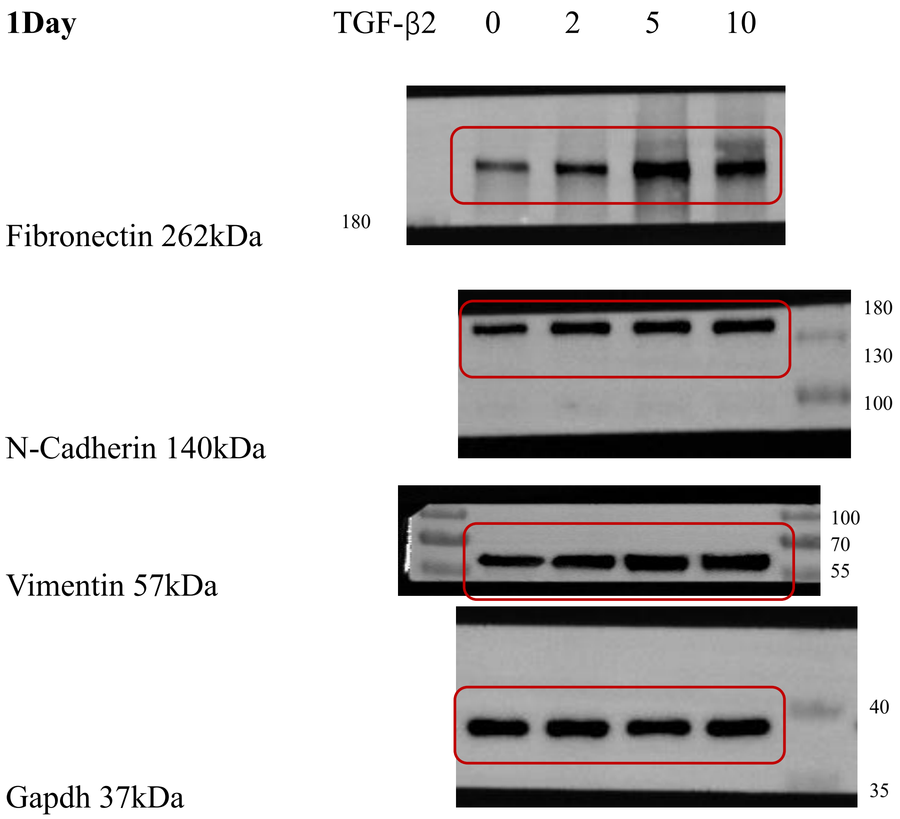

Full length membranes without markers:

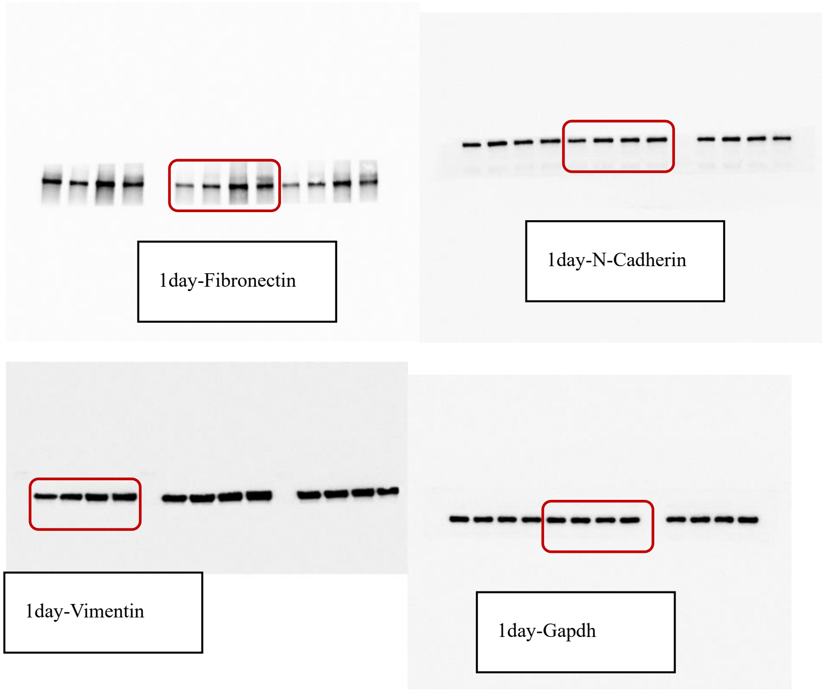

Fig S3. The original figure before being cropped in Fig 1A.

2Day TGF-β2 0 2 5 10

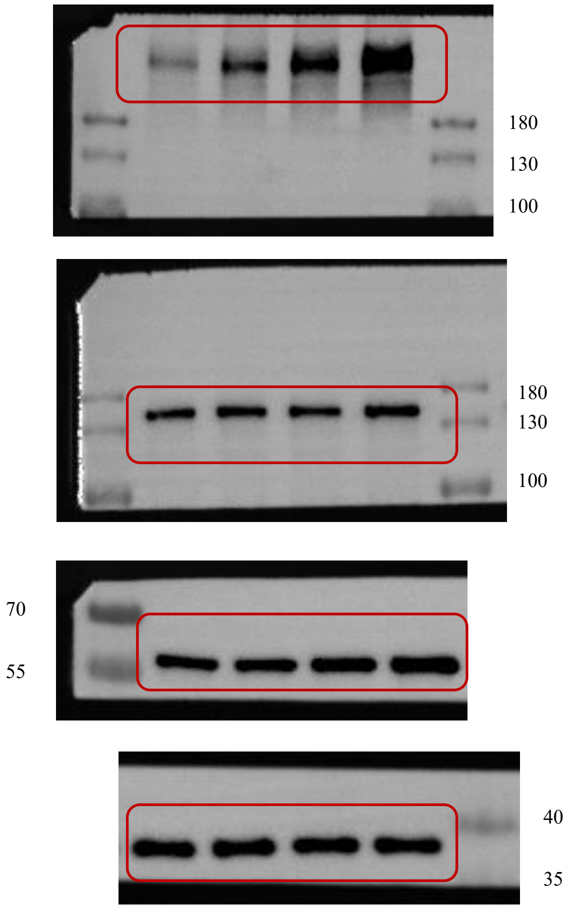

Fibronectin 262kDa

N-Cadherin 140kDa

Vimentin 57kDa

Gapdh 37kDa

Full length membranes without markers:

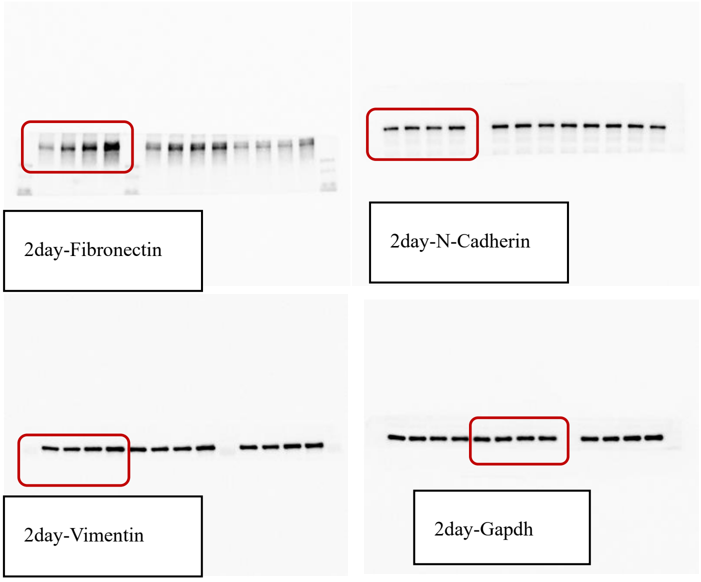

Fig S3. The original figure before being cropped in Fig 1A.

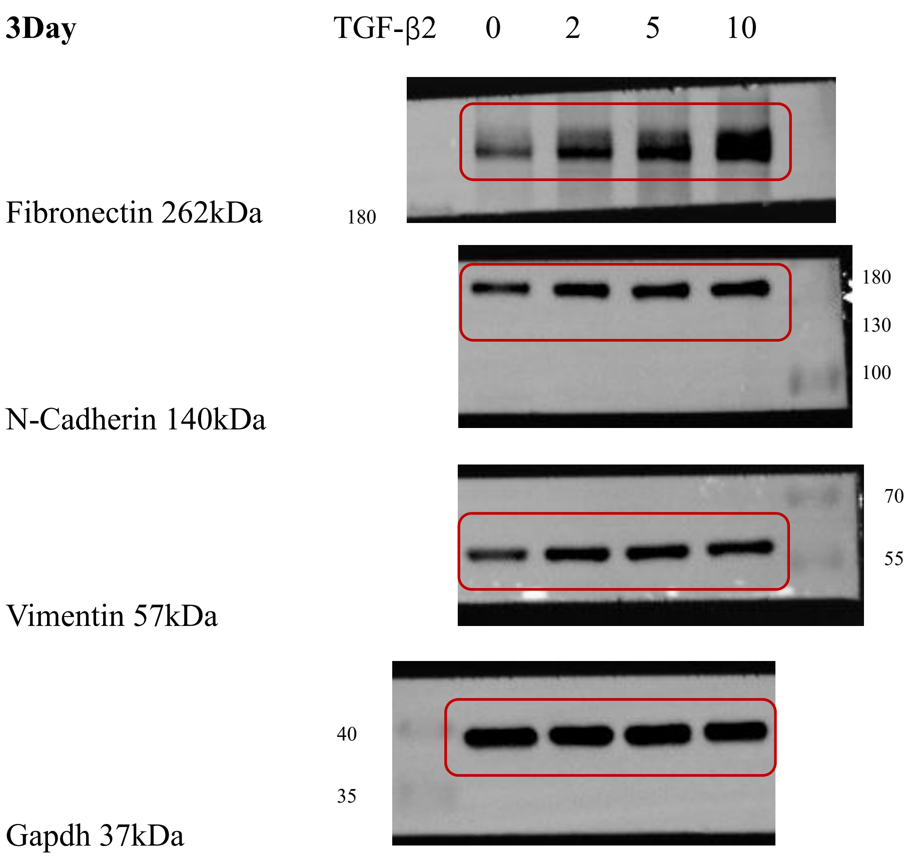

Full length membranes without markers:

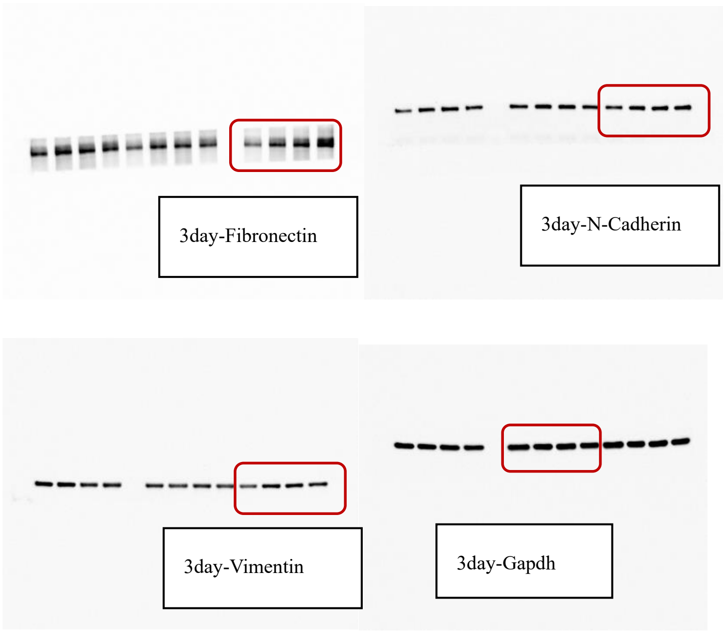

Fig S3. The original figure before being cropped in Fig 1A.

The samples in the same day derived from the same experiment and the blots were processed in parallel. The blots were cut prior to hybridization with primary antibodies during western blotting.

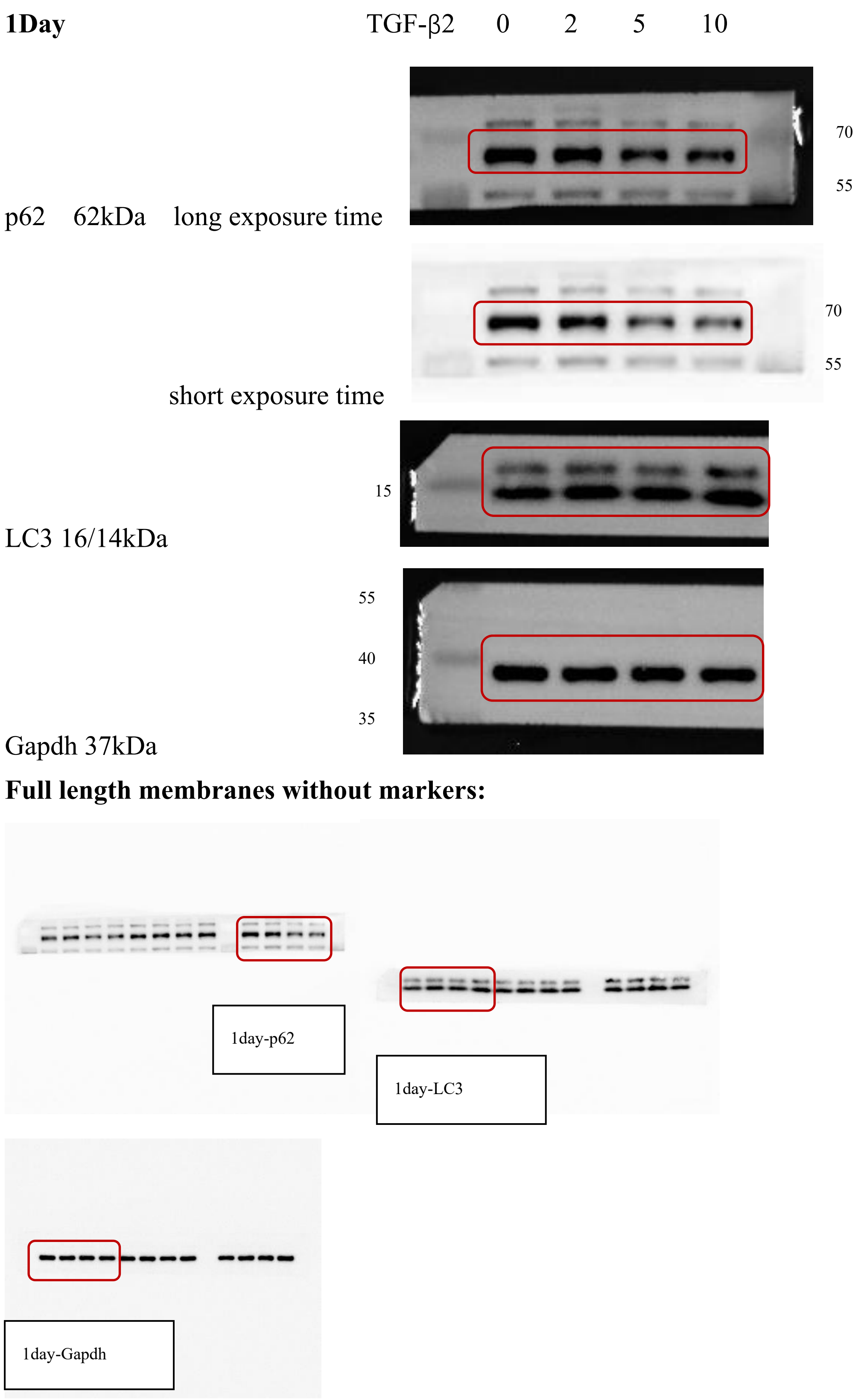

Fig S4. The original figure before being cropped in Fig 1C.

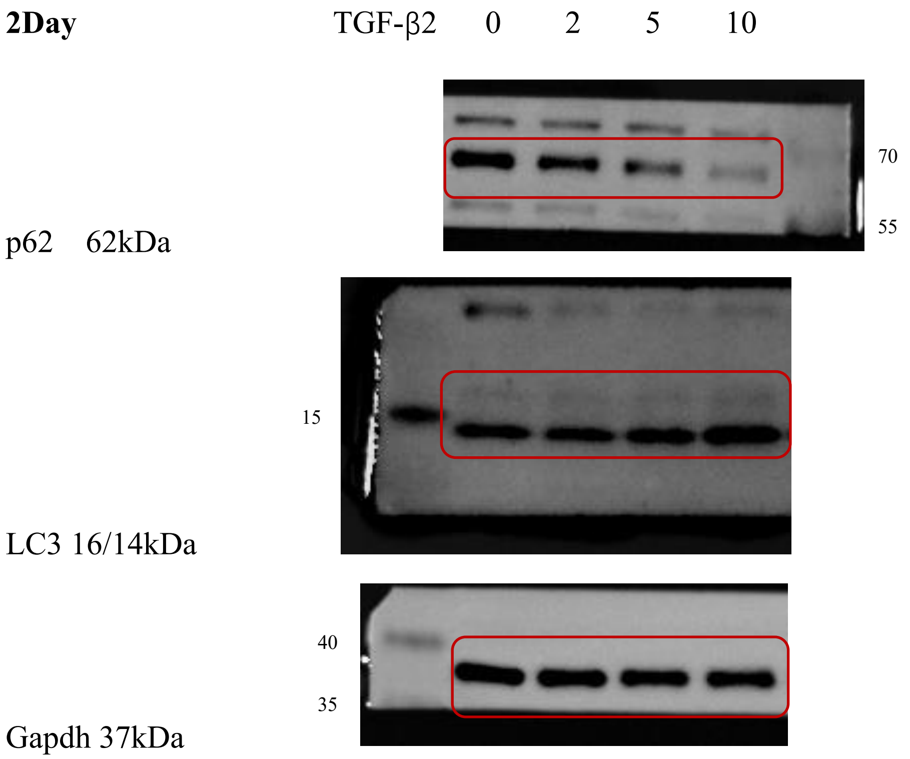

Full length membranes without markers:

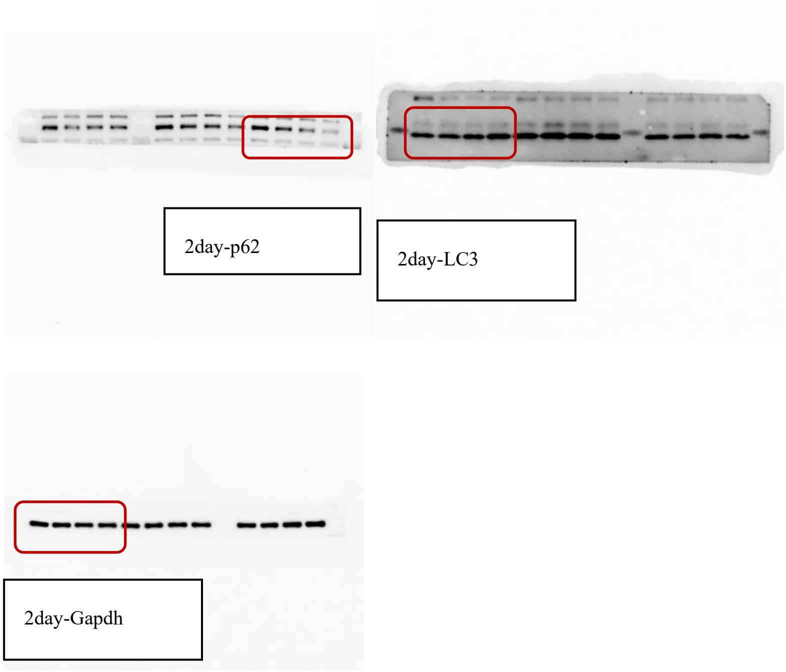

Fig S4. The original figure before being cropped in Fig 1C.

3Day TGF-β2 0 2 5 10

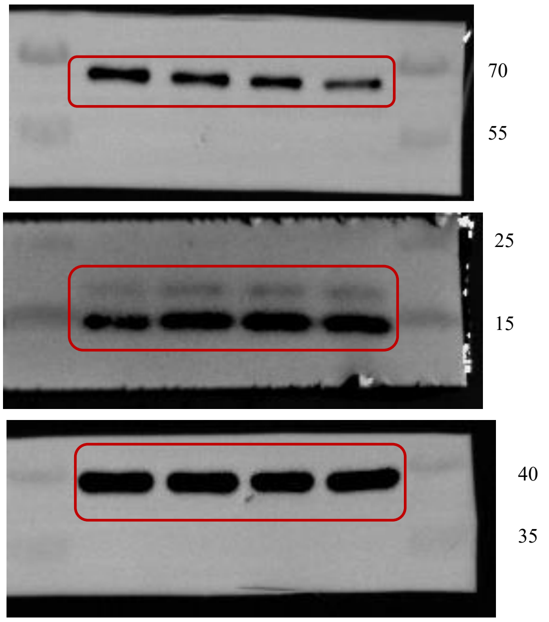

p62 62kDa

LC3 16/14kDa

Gapdh 37kDa

Full length membranes without markers:

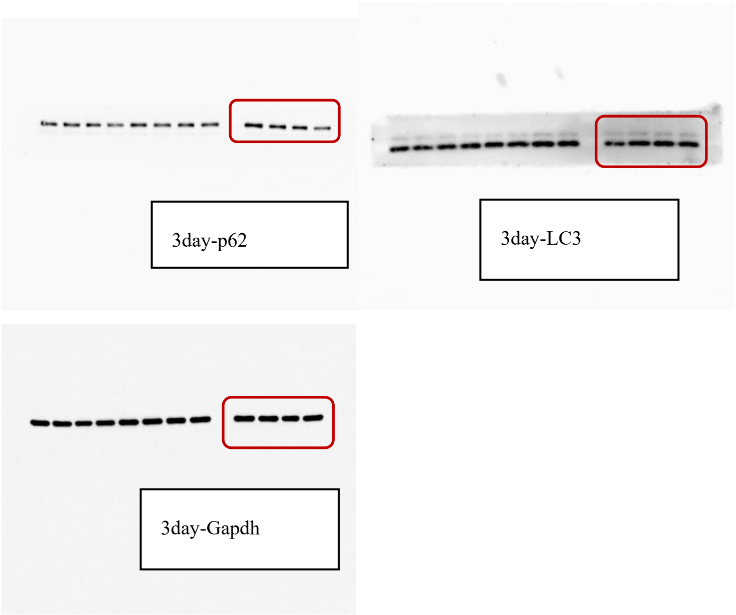

Fig S4. The original figure before being cropped in Fig 1C.

The samples in the same day derived from the same experiment and the blots were processed in parallel. The blots were cut prior to hybridization with primary antibodies during western blotting.

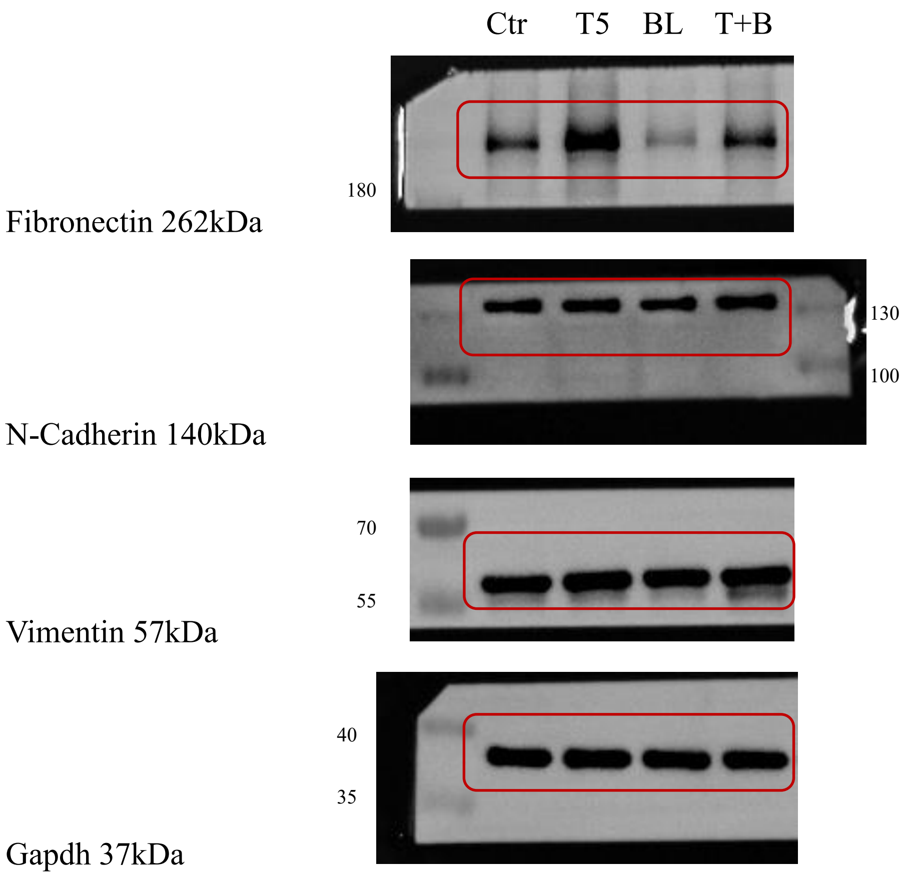

Full length membranes without markers:

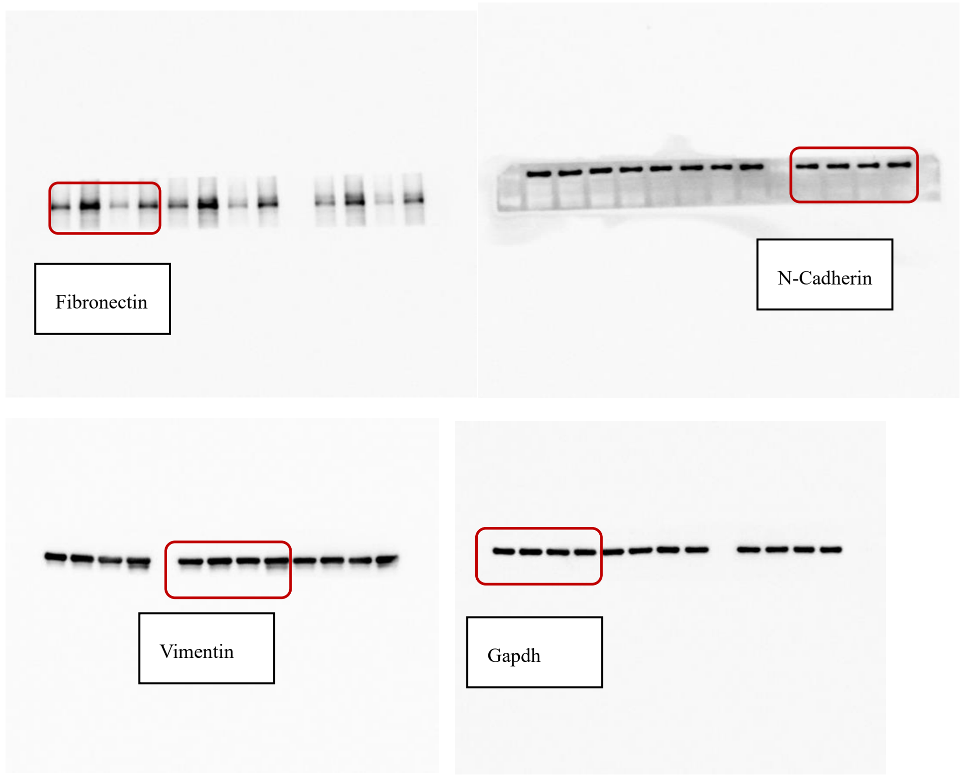

Fig S5. The original figure before being cropped in Fig 2A.

The samples in the same day derived from the same experiment and the blots were processed in parallel. The blots were cut prior to hybridization with primary antibodies

during western blotting.

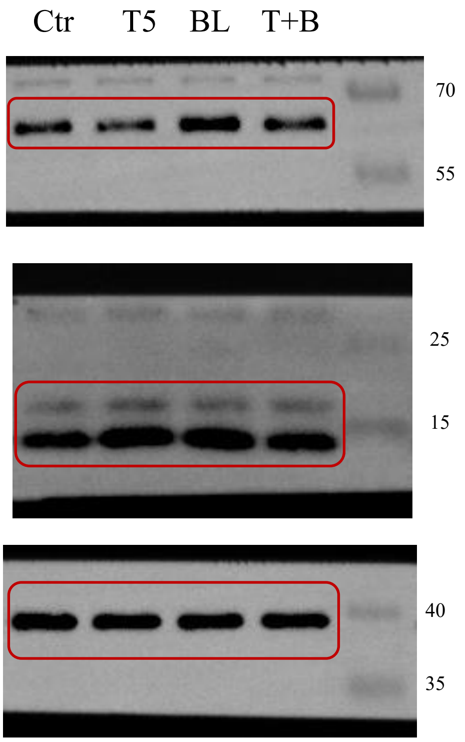

p62 62kDa

LC3 16/14kDa

Gapdh 37kDa

Full length membranes without markers:

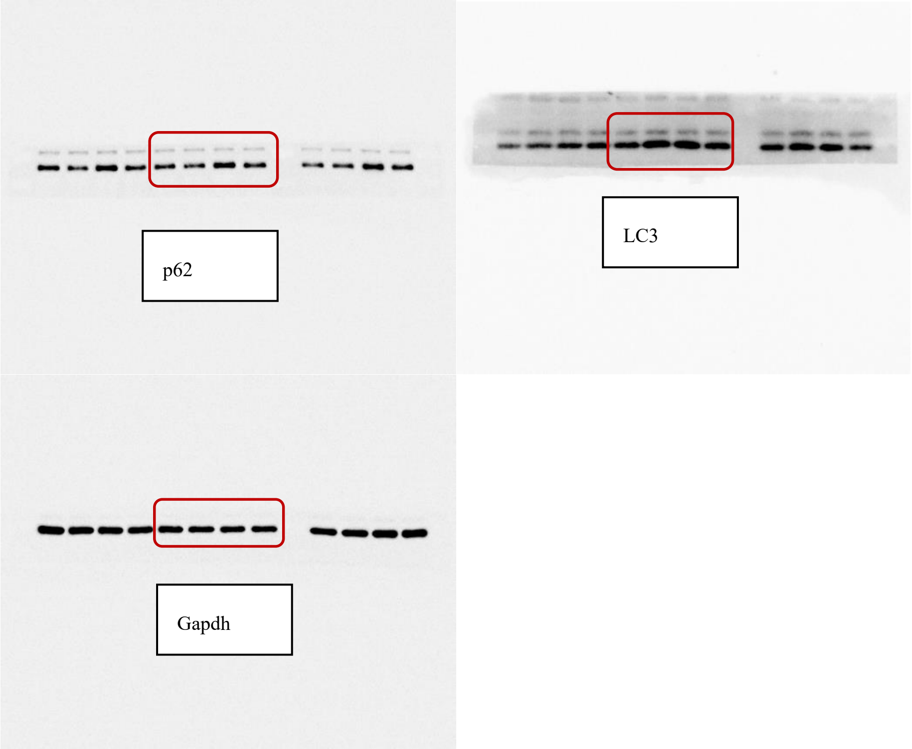

Fig S6. The original figure before being cropped in Figure 3A.

The samples in the same day derived from the same experiment and the blots were processed in parallel. The blots were cut prior to hybridization with primary antibodies during western blotting.

Fig 5A:

T5	-	-	+	+	-	-	+	+
BL	-	-	-	-	+	+	+	+
Rapa	-	+	-	+	-	+	-	+

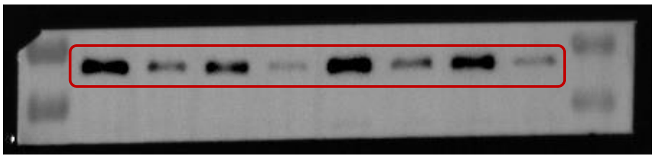

p62 62kDa

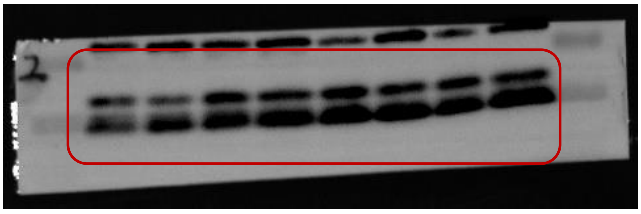

LC3 16/14kDa

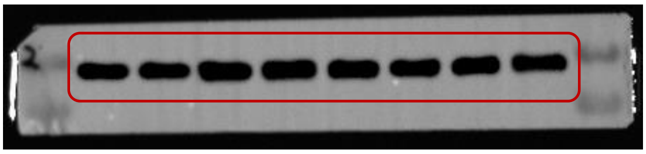

Gapdh 37kDa

Full length membranes without markers:

p62:

LC3: 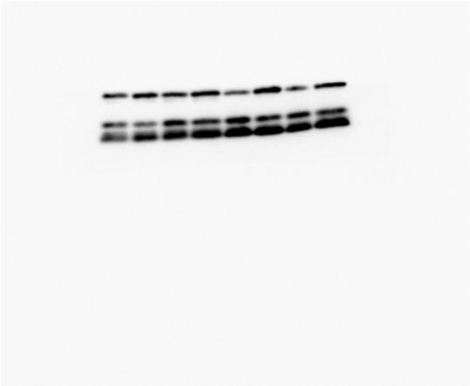

Gapdh: 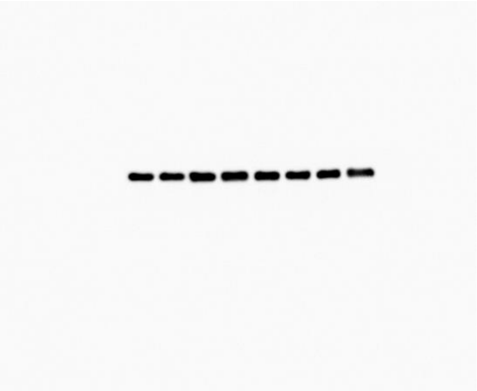

Fig S7. The original figure before being cropped in Figure 5A.

The samples in the same day derived from the same experiment and the blots were processed in parallel. The blots were cut prior to hybridization with primary antibodies during western blotting.

Fig 5B:

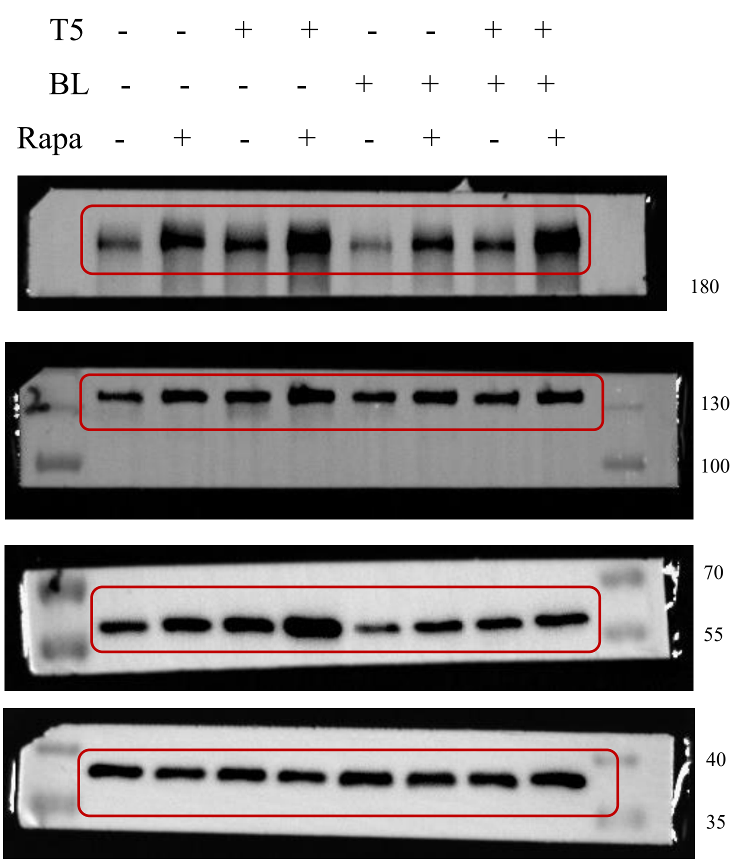

Fibronectin 262kDa

N-Cadherin 140kDa

Vimentin 57kDa

Gapdh 37kDa

Full length membranes without markers:

Fibronectin:

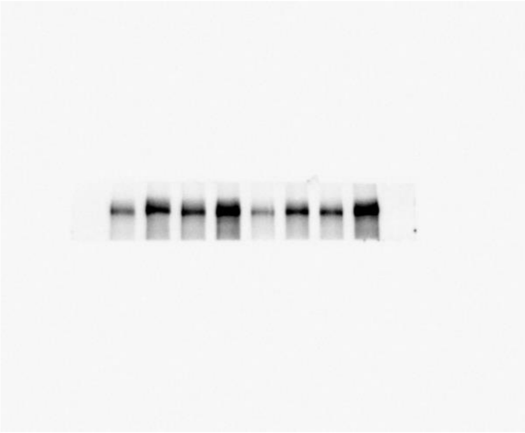

N-Cadherin:

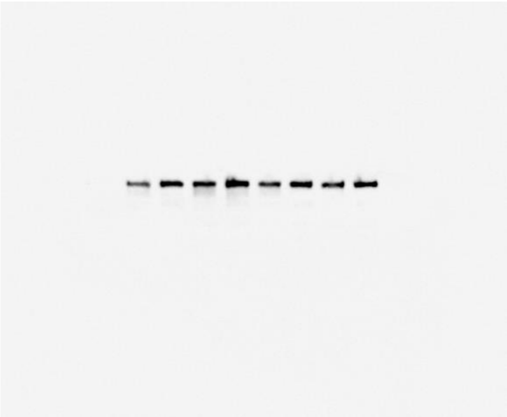

Vimentin: 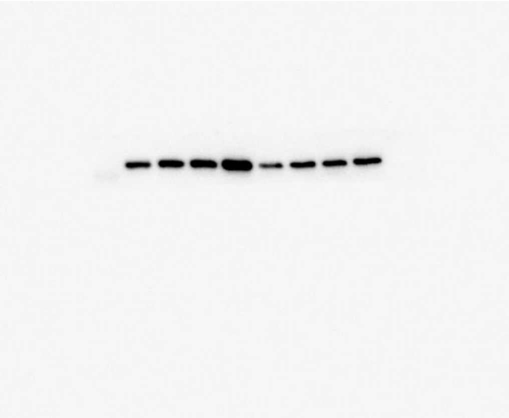

Gapdh: 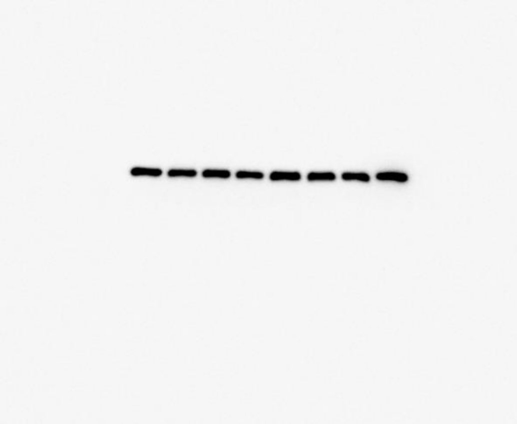

Fig S8. The original figure before being cropped in Figure 5B.

The samples in the same day derived from the same experiment and the blots were processed in parallel. The blots were cut prior to hybridization with primary antibodies during western blotting.

Fig 6A:

T5	-	-	+	+	-	-	+	+
BL	-	-	-	-	+	+	+	+
CQ	-	+	-	+	-	+	-	+

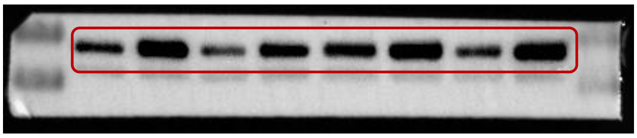

p62 62kDa

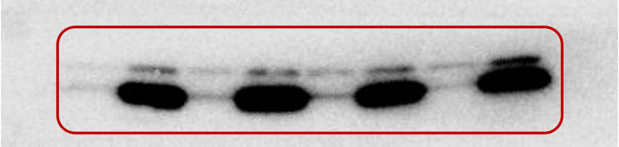

LC3 16/14kDa

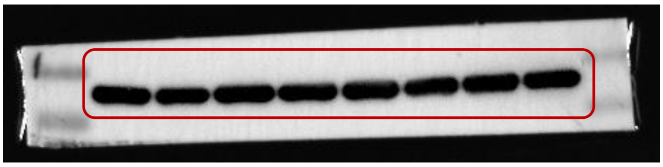

Gapdh 37kDa

Full length membranes without markers:

p62: 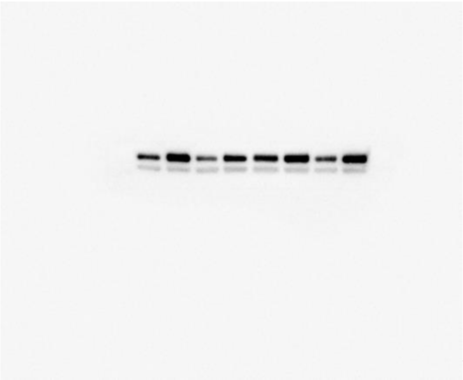

LC3: 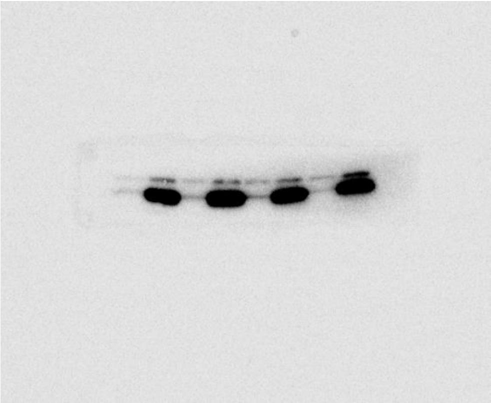

Gapdh: 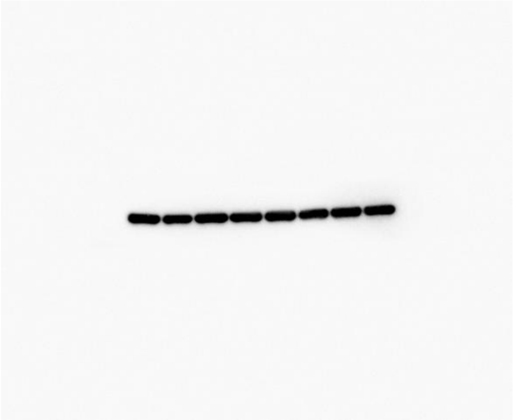

Fig S9. The original figure before being cropped in Figure 6A.

The samples in the same day derived from the same experiment and the blots were processed in parallel.

Fig 6B:

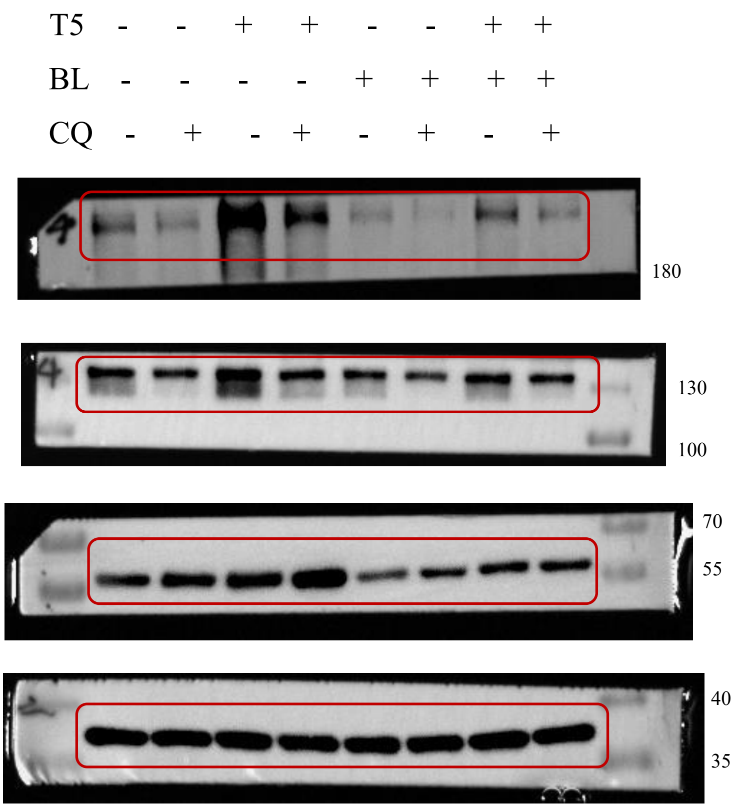

Fibronectin 262kDa

N-Cadherin 140kDa

Vimentin 57kDa

Gapdh 37kDa

Full length membranes without markers:

Fibronectin:

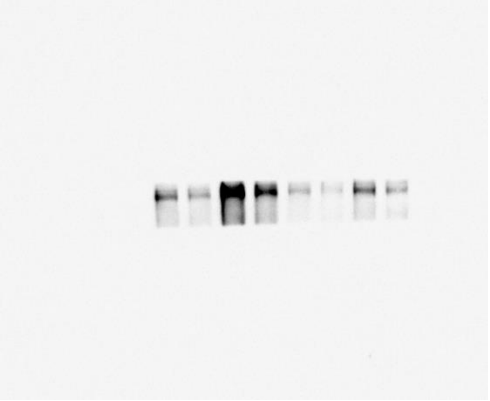

N-Cadherin:

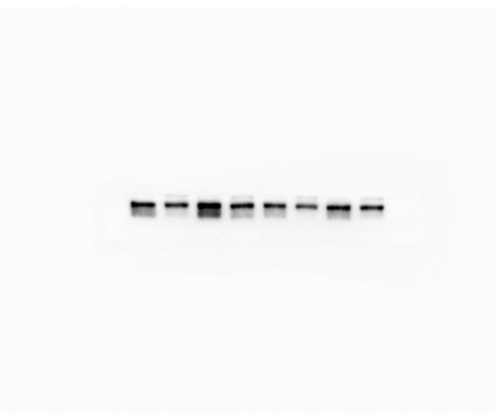

Vimentin: 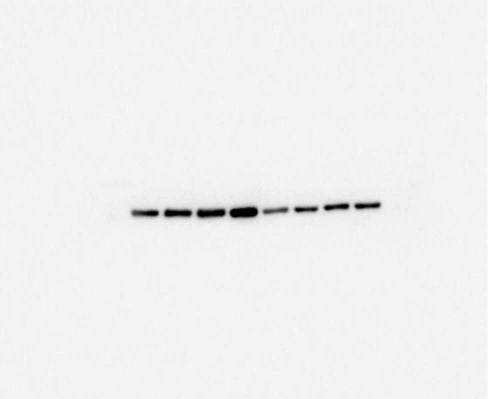

Gapdh: 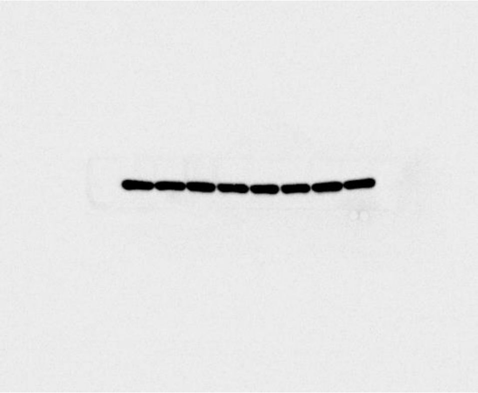

Fig S10. The original figure before being cropped in Figure 6B.

The samples in the same day derived from the same experiment and the blots were processed in parallel.
